# Bone health in spacefaring rodents and primates: systematic review and meta-analysis

**DOI:** 10.1038/s41526-021-00147-7

**Published:** 2021-06-01

**Authors:** Jingyan Fu, Matthew Goldsmith, Sequoia D. Crooks, Sean F. Condon, Martin Morris, Svetlana V. Komarova

**Affiliations:** 1 Shriners Hospitals for Children - Canada, Montréal, Canada; 2 Faculty of Dentistry, McGill University, Montréal, Canada; 3 Schulich Library of Physical Sciences, Life Sciences and Engineering, McGill University, Montréal, Canada

**Keywords:** Physiology, Computational biology and bioinformatics

## Abstract

Animals in space exploration studies serve both as a model for human physiology and as a means to understand the physiological effects of microgravity. To quantify the microgravity-induced changes to bone health in animals, we systematically searched Medline, Embase, Web of Science, BIOSIS, and NASA Technical reports. We selected 40 papers focusing on the bone health of 95 rats, 61 mice, and 9 rhesus monkeys from 22 space missions. The percentage difference from ground control in rodents was –24.1% [Confidence interval: −43.4, −4.9] for trabecular bone volume fraction and –5.9% [−8.0, −3.8] for the cortical area. In primates, trabecular bone volume fraction was lower by –25.2% [−35.6, −14.7] in spaceflight animals compared to GC. Bone formation indices in rodent trabecular and cortical bone were significantly lower in microgravity. In contrast, osteoclast numbers were not affected in rats and were variably affected in mice. Thus, microgravity induces bone deficits in rodents and primates likely through the suppression of bone formation.

## Introduction

With plans by NASA to return humans to the lunar surface by 2024^1^ and to have the first-ever astronauts journey to Mars within the next 2 decades^2^, in addition to private interests in developing the first human colony on the Martian surface^3^, human space travel will no doubt continue if not increase in the following century. Despite these high ambitions, we still do not fully understand the cause of physiological changes we observe in astronauts who travel to space, one of which is microgravity-induced bone loss^4,5^.

Animals have long been used as models to assess the physiological changes observed as a result of various stimuli and inform their impact on human health. Space-traveling animals have even preceded humans, with several dogs, rodents, and primates being sent to space in the late 1940s–1960s^6^. After developing the necessary technology allowing mammals to survive all phases of spaceflight, beginning in the 1970s animal experiments shifted to focus on the physiological effects of space travel^7^. The information obtained in animal studies significantly augmented our knowledge regarding human adaptations during space travel. Experiments assessing skeletal changes in animals have the benefit of the collection of bone biopsies, which is absent in astronaut studies. These biopsies have allowed for an investigation into changes to cellular and molecular components of bone associated with microgravity, and thus provide further insight into the underlying mechanisms of microgravity-induced changes in bone health. These missions however come at a considerable price, and it has been estimated that NASA spent $1.2 billion per launch over the period from 1982 to 2010^8^, therefore it is critically important to gain as much knowledge as possible from all the space experiments.

Even with the benefits of animal studies, and a significant expense associated with their execution, these experiments have not yet been used for the purposes of quantitative data synthesis. To overcome the problems associated with small sample sizes and a high degree of variability between individual missions we employed meta-analysis to improves the statistical power of all the studies. Thus, the objectives of this study were to (i) to systematically identify all the published literature regarding bone health in vertebrate animals that were part of experiments performed in space; (ii) use a meta-analytic approach to quantitatively characterize space-related changes to bone architecture and turnover in animals, (iii) identify cofounding variables associated with changes in bone health.

## Results

### Overview of relevant studies

**Fig. 1:**
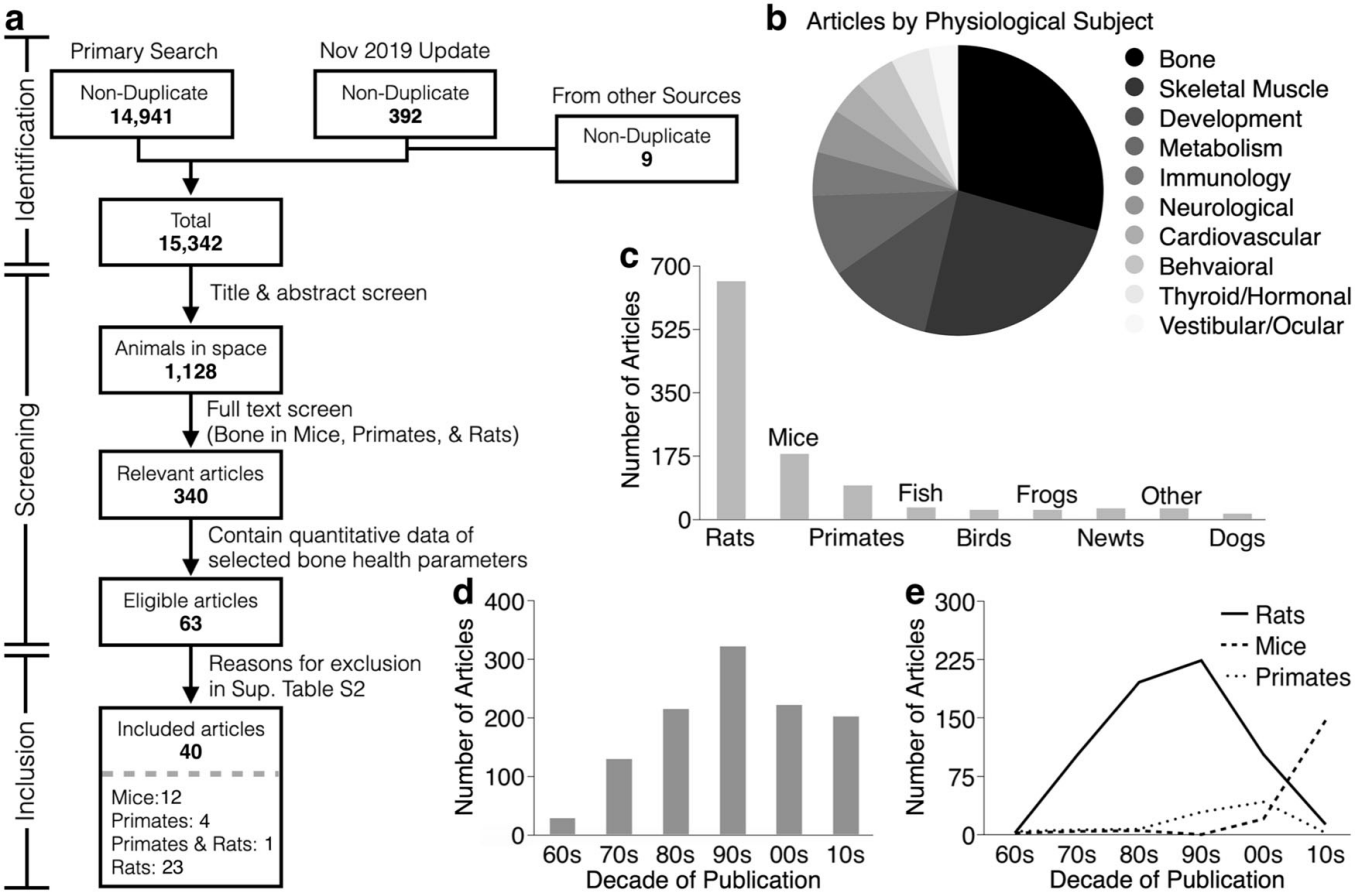
Systematic review information flow and outcomes. **a** Prisma diagram. **b**–**e** Analysis of 1128 articles selected after the title and abstract screening. **b** Distribution of physiological systems mentioned in the papers. **c** The number of articles discussing indicated species. **d** The number of articles by publication decade. **e** The number of articles by publication decade for species of rats (solid line), mice (dashed line), and primates (dotted line).

The systematic search describing the overlap of space travel, animals, and bone executed in Medline, Embase, Web of Science, and BIOSIS, together with the 9 reports found via manual searches of the NASA Technical Report Server and the compendium of animal and cell spaceflight experiments compiled by Ronca et al.^9^ resulted in the identification of 1128 candidate articles (Fig. 1a). Of these, 340 articles focused on bone, while the rest discussed a range of physiological systems potentially relevant to bone health, including skeletal muscles, metabolism, and developmental issues (Fig. 1b). The majority of studies (83%) described findings in rats (664/1128), mice (181/1128), and primates (96/1128) (Fig. 1c). The number of papers describing animals in space peaked in the 1990s (Fig. 1d). From the 1970s through the 2000s, rats were the main spacefaring animal model. Interest in primates peaked in the 2000s, however, in the last decade mice have become the predominant animal model studied in space (Fig. 1e). Considering the available data, the full-text screen focused on 340 studies describing bone health in rodents and primates and identified 63 studies that presented quantitative measurements of trabecular and cortical bone architecture or bone turnover (Table 1)^10–72^. After excluding studies that reported data on treated animals, reported duplicate data, or demonstrated unclear reporting (Supplementary Table 3), 40 articles were selected for the final meta-analysis: 23 describing rats^10–32^, 12 describing mice^33–44^, 4 describing primates^45–48^, and 1 describing both mice and primates^49^. The final dataset included a total of 95 rats, 61 mice, and 9 primates (rhesus macaque monkeys) flown to space on 22 missions (Table 2).

**Table 1:** Bone parameters included in the meta-analysis.

Parameter (abbreviation)	Description	Units
Trabecular (Tb) bone measures		
1. Tb. bone volume fraction (Tb.BV/TV)	Fraction of the cancellous space occupied by Tb bone	%
2. Tb. Thickness (Tb.Th)	Mean thickness of trabeculae	mm or μm
3. Tb. Number (Tb.N)	Mean number of trabeculae per unit length	mm^−1^
4. Tb. Separation (Tb.Sp)	Mean distance between trabeculae	mm or μm
5. Connectivity density	Number of connected trabeculae per unit volume	mm^−3^
6. Total bone volume fraction (Total BV/TV)	Total bone volume/tissue volume	%
Cortical (Ct.) bone measures		
1. Marrow Area (Ma.Ar)	Cross-sectional area occupied by medullary canal	mm^2^
2. Marrow Diameter (Ma.Dm)	Mean diameter of medullary canal	mm
3. Ct. Thickness (Ct.Th)	Cross-sectional thickness of cortical bone	mm or μm
4. Ct. Bone Area (Ct.Ar)	Cross-sectional area occupied by cortical bone	mm^2^
Bone turnover measures		
1. Osteoblast Surface (Ob.S/BS)	Percent of bone surface covered with osteoblasts	%
2. Osteoblast Number (N.Ob)	Number of osteoblasts per length of bone^15,19^ or per visual field^17^	#/mm or #/field
3. Osteoid Surface (OS/BS)	Percentage of bone surface covered in osteoid	%
4. Osteoid Thickness (O.Th)	Mean thickness of osteoid seams	μm
5. Osteoclast Surface (Oc.S/BS)	Percent of bone surface covered with osteoclasts	%
6. Osteoclast Number (N.Oc)	Number of osteoclasts per length of bone^19,24,28^ or per visual field^17^	#/mm or #/field
7. Bone Formation Rate (BFR)	Volume^12,14,33^ or area^19,26,30,32^ of bone formed per day, normalized to bone volume^33^ or bone length^32^	mm^3^/day mm^2^/day %/day mm^2^/mm/day
8. Mineral Apposition Rate (MAR)	Thickness of new bone formed per day	μm/day

**Table 2:** Description of articles included in the meta-analysis.

Year	Mission	Days	Article	Species	*n*_SF_	Type of Control	Bones analyzed (sub-sections)	QS (/25)
1975	Cosmos 782	19.5	Asling^10^	Rats	6	GC, VC	Tibia (M)	13^a^
			Morey^11^		11	GC, VC	Tibia (D)	20
1977	Cosmos 936	18.5	Morey-Holton^12^	Rats	10	GC, VC	Tibia (D)	18^a^
1979	Cosmos 1129	18.5	Judy^13^	Rats	7	VC	Tibia (M)	14^a^
			Wronski^14^		11	GC, VC	Rib(NS), Humerus (D), Tibia (D)	20^a^
			Jee^15^		7		Humerus (M), Tibia (M)	19
			Rogacheva^16^		6		Femur (D)	12.5
1983	Cosmos 1514	5	Cann^45^	Primate	1	GC	Ulna (D), Radius (D), Tibia (D)	14.5^a^
1985	Cosmos 1667	7	Kaplanskii^17^	Rats	7	GC, VC	*Vertebrae (L)*, Pelvis (Ilium), Tibia (D, *M*)	15.5
			Vico^18^			GC	Vertebrae (T8, L1), Femur (M), Tibia (M)	17
1985	SpaceLab3	7	Wronski^19^	Rats	L5 S6	GC	Vertebra (L4), Humerus (M), Tibia (D)	19.5
1987	Cosmos 1887	12.5	Vailas^21^	Rats	5	GC, VC	Humerus (D)	20
			Doty^20^				Tibia (D)	19
			Zerath^22^			VC	Vertebrae (NS), Humerus (M)	14
			Cann^46^	Primates	2	DS, VC	Ulna (D), Radius (D), Tibia (D)	16.5^a^
1989	Cosmos 2044	14	Zerath^49^	Primates	2	DS	Pelvis (Ilium)	15.5
				Rats	5	GC, VC	Vertebra (T9), Humerus (M)	18
			Vailas^21^				Humerus (D)	19
			Vico^24^				Vertebra (L2, T5), Femur (M), Tibia (E,M)	20
1992	Bion 10	11.5	Zerath^47^	Primates	2	DS, GC, VC	Pelvis (Ilium)	20
1992	STS-52	10	Turner^25^	Rats	6	GC	Humerus (M)	16
1992	STS-57	11	Westerlind^26^	Rats	12	GC, VC	Femur (D), Tibia (M)	23
1993	STS-58 (SLS-2)	14	Zerath^27^	Rats	5	GC, VC	Vertebrae (T9,C7), Humerus (M)	23
			Lafage-Proust^28^				Femur (M), *Humerus (M)*	18
1996	Bion 11	14	Zerath^48^	Primates	2	GC, VC	Pelvis (Ilium)	21
1996	STS-77	10	Bateman^29^	Rats	6	VC	Humerus (D), Tibia (D)	20
1996	STS-78	17	Wronski^30^	Rats	6	GC, VC	Vertebra (L1), Tibia (M, D)	24
			Zerath^31^				Vertebra (T8), Pelvis (Cotyloid)	22
			Vajda^32^				Femur (D)	21
2001	STS-108	12	Lloyd^33^	Mice	12	GC	Vertebra (L5), Humerus (M), Femur (D), Tibia (M)	21
2007	STS-118	13	Ortega^34^	Mice	12	GC	Femur (M), Tibia (M, D)	18.5
2010	STS-131	15	Blaber^35^	Mice	7	GC	Pelvis (Ischium), Femur (M)	19
			Zhang^36^				Calvaria	16.5
			Blaber^37^		8		Femur (E, M)	17.5
2013	Bion M1	30	Berg-Johansen^38^	Mice	3	VC	Vertebrae (C)	16
			Macaulay^39^		6	GC	Calvaria	21
			Gerbaix^40^		5		Vertebrae (L1,L3,T12), Femur (M,D)	20
			Gerbaix^41^				Calcaneus, Navicular, Talus	17
2016	SpaceX CRS-9	39	Shiba^42^	Mice	5	GC	Femur (prox)	17.5
2017	SpaceX CRS-10	28	Maupin^43^	Mice	10	GC	Calvaria, Rib (10), Sternum, Vertebra (L4), Humerus (M,D), Femur (M,D), Tibia (M,D)	21
2017	SpaceX CRS-12	34	Tominari^44^	Mice	3	GC	Humerus (prox), Tibia (prox)	17

Days*:* mission duration (days); *n*_SF_: sample size of spaceflight animal group.

Control groups: *GC* ground control, *VC* vivarium control, *DS* delayed stimulation.

Sub-sections of bones analyzed: *E* epiphysis, *M* metaphysis, *D* diaphysis, prox proximal.

For vertebrae region: *L* lumber, *T* thoracic, *C* caudal, *NS* not specified.

Italics indicate overlapping bones measured excluded from the meta-analysis. QS = quality score calculated according to Supplementary note 2.

^a^Indicates articles sourced from NASA Final Reports of Soviet missions. For the specific measurements present in each study, refer to Supplementary Table 4. For rodent study characteristics used for covariate analysis, refer to Supplementary Table 5.

### Heterogeneity, bias, and the meta-analytic model

**Fig. 2:**
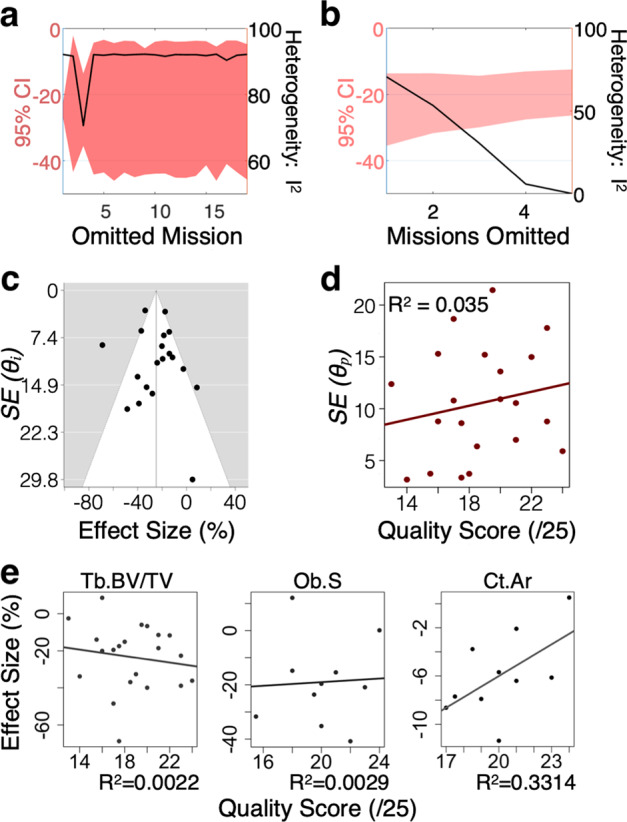
Heterogeneity and sensitivity analyses for the BV/TV dataset. **a**, **b** Heterogeneity was analyzed using single mission exclusion (**a**) and cumulative mission exclusion (**b**). Red area: 95% CI for the global effect size (left axis); line: *I*^*2*^ (right axis). **c** Funnel plot. (**d**) Article-level standard error SE *(θ*_*p*_*)* as a function of quality score. **e** Meta-regression of the Tb.BV/TV, Ob.S, and Ct.Ar paper-level outcomes as a function of quality score. The maximum quality score was 25. *R*^2^ is shown.

Statistical heterogeneity was moderate to high (*I*^*2*^ > 46%) for all the extracted parameters for spaceflight-related changes except for bone marrow area (*I*^*2*^ = 14.4%) and cortical bone area (*I*^*2*^ = 0%). Single mission exclusion analysis identified some mission-level outcomes removing which reduced the overall heterogeneity, however, no single mission influenced the heterogeneity of more than one parameter or the global outcome for Tb.BV/TV or Tb.N parameter datasets (Fig. 2a, Supplementary Fig. 1a). Cumulative-mission exclusion analysis demonstrated that exclusion of >21% of missions led to a homogeneous (*I*^*2*^ ≤ 30%) dataset, and that the overall outcomes for Tb.BV/TV and Tb.N were not affected by decreased heterogeneity (Fig. 2b, Supplementary Fig. 1b). The funnel plot demonstrated symmetrical distribution (Fig. 2c, Supplementary Fig. 1c). We assessed the quality of individual papers on a 25-point scale (Supplementary note 2) and examined if quality score affected the reported paper-level variance (Fig. 2d, Supplementary Fig. 1d) or effect size (Fig. 2e), however, no significant association of quality score with reported outcomes was observed. Subgroup analysis further demonstrated no difference between papers with low (<20) and high (≥20) quality scores (Supplementary Fig. 2). We conclude that the publication bias is negligible within this dataset. To account for low sample sizes, as well as heterogeneity, we used the modified sampling by size method^73^ for further analysis.

### Changes in trabecular parameters during spaceflight

**Fig. 3:**
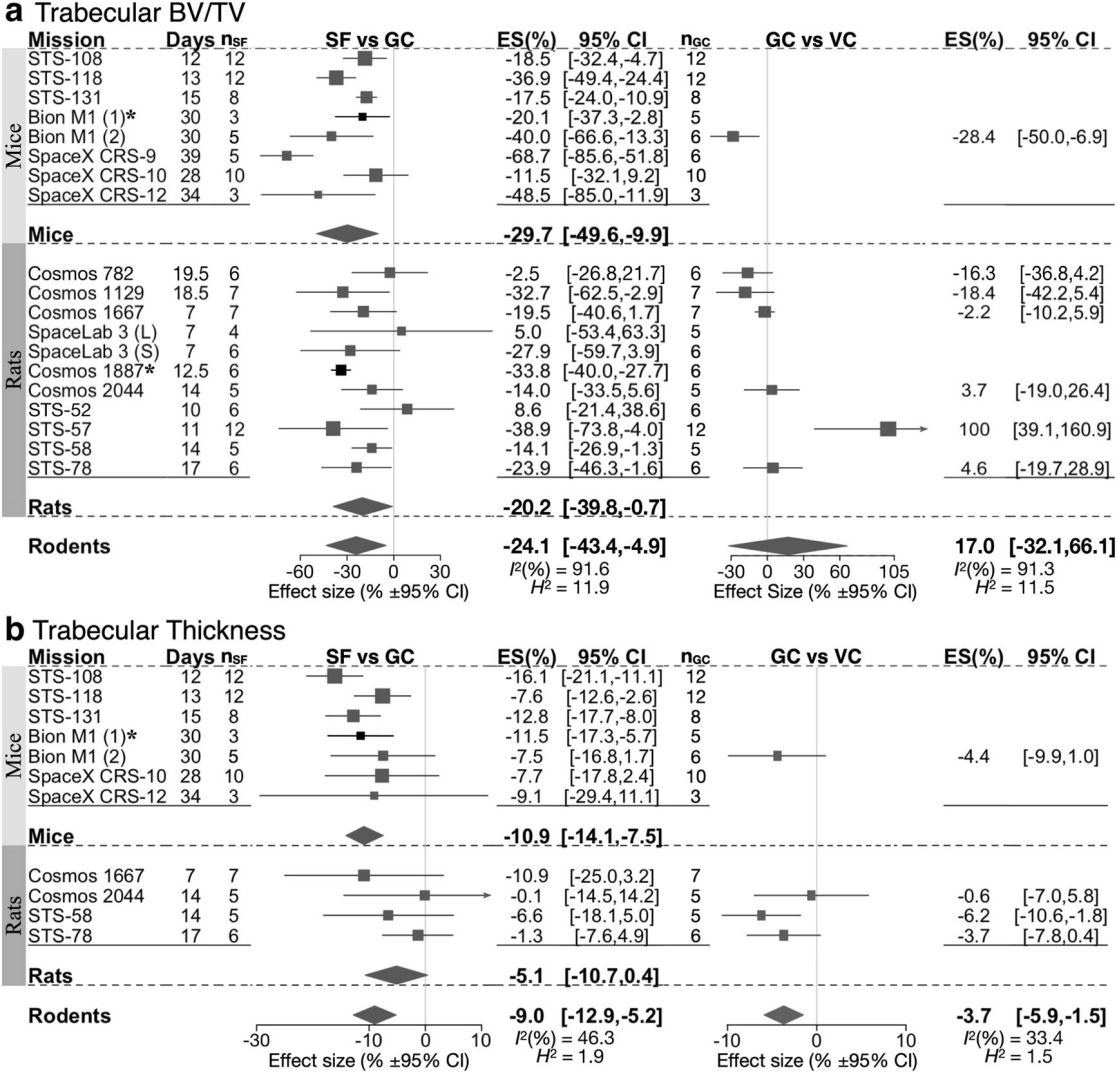
Forest plot of spaceflight and ground control-induced changes to Tb.BV/TV and trabecular thickness. Changes in BV/TV (**a**) and trabecular thickness (**b**) of spaceflight animals (SF) compared to ground control animals (GC) (Left); and GC compared to vivarium control animals (VC) (Right). For each indicated species, missions are sorted by mission year (old to new); duration of spaceflight (Days), and the number of spaceflight animals (*n*_SF_) are indicated. Square/line: effect size (%) and 95% CI, the size of the square is proportional to *n*_SF_. Overall effect size (%) and 95% CI are indicated by diamonds for mice, rats, and rodents, *I*^*2*^, and *H*^2^ are given for rodents. Asterisk (*) indicates missions wherein GC was not present, and SF was compared to VC.

**Fig. 4:**
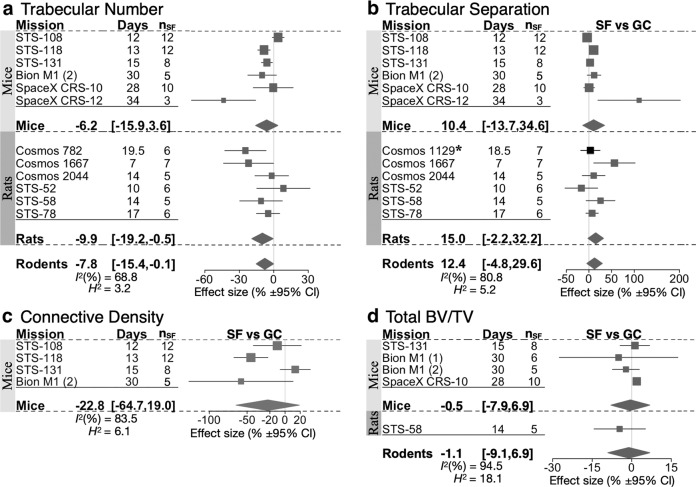
Forest plot of spaceflight induced changes to the trabecular number, trabecular separation, connective density, and total BV/TV. Changes in trabecular number (**a**), trabecular separation (**b**), connective density (**c**) and total BV/TV (**d**) of space flight animals (SF) compared to ground control animals (GC). For each indicated species, missions are sorted by mission year (old to new); duration of spaceflight (Days), and the number of spaceflight animals (*n*_SF_) are indicated. Square/line: effect size (%) and 95% CI, the size of the square is proportional to *n*_SF_. Overall effect size (%) and 95% CI are indicated by diamonds for mice, rats, and rodents, *I*^*2*^, and *H*^2^ are given for rodents. Asterisk (*) indicates missions wherein GC was not present, and SF was compared to VC. For mission-level effect sizes and 95% CI, refer to Supplementary Tables 6–9.

Many studies included two types of control—vivarium control (VC), where animals lived in a standard laboratory habitat, and the ground control (GC), where some or all aspects of spaceflight other than microgravity, such as physical enclosure, diet and lift off and re-entry forces, were simulated. We examined the percentage difference in spaceflight compared to GC, as well as in GC compared to VC. Of the 6 parameters describing trabecular bone: trabecular bone volume fraction (Tb.BV/TV), thickness (Tb.Th), number (Tb.N), separation (Tb.Sp), connective density, and Total BV/TV; Tb.BV/TV was significantly lower in spaceflight mice and rats compared to ground control, and Tb.Th was significantly reduced for mice (Fig. 3a, b left). For rodents overall, Tb.BV/TV and Tb.Th changed significantly by −24.1% [−43.4, −4.9] and −9.0% [−12.9, −5.2], respectively. Tb.N, Tb.Sp, and connective density demonstrated trends towards poor bone health in spaceflight mice and rats, however only the change in Tb.N reached statistical significance (Fig. 4a–c). Total BV/TV, which was measured in flat bones and in one case vertebra, did not change due to spaceflight (Fig. 4d). When ground and vivarium controls were compared, Tb.BV/TV, Tb.N, and Tb.Sp were unaffected, but Tb.Th was significantly lower in GC compared to VC (Fig. 3a, b right, Supplementary Tables 6, 7), suggesting that flight conditions other than microgravity may contribute to a reduced Tb.Th. In all trabecular parameters in rodents, heterogeneity was moderate to high, *I*^*2*^ > 46%. Trabecular parameters were measured in 4 primates on missions Bion 10 and 11, and demonstrated significantly lower Tb.BV/TV, a trend to reduced Tb.N, and Tb.Th, and a trend to higher Tb.Sp compared to GC (Table 3). Thus, there was a deficit in trabecular bone in rodents and primates after the spaceflight.

**Table 3:** Spaceflight-induced changes in bone parameters of primates.

Missions	Σ*n*_*SF*_/Σ*n*_*GC*_	SF vs. GC		GC vs. VC	
		ES (%)	95% CI	ES (%)	95% CI
Tb.BV/TV					
Bion 10 Bion 11	4/7	−25.2	[−35.6, −14.7]	−4.6	[−27.4, 18.3]
Tb.Th					
Bion 10 Bion 11	4	−14.7	[−34.1, 4.8]	–	–
Tb.N					
Bion 10 Bion 11	4	−7.8	[−16.9, 1.4]	–	–
Tb.Sp					
Bion 10 Bion 11	4	8.5	[−12.3, 29.2]	–	–
Ct.Th					
Cosmos 1514 Cosmos 1887^a^ Bion 11	5/4	−6.4	[−84.2, 71.4]	0.8	[−5.4, 7.0]
MAR					
Bion 10 Bion 11 Cosmos 2044^a^	6/10	−31.4	[−62.0, −0.7]	1.8	[−2.7, 6.2]

^a^missions in which ground control (GC) was not a present and delayed simulation (DS) was used as GC, which were also excluded from GC vs. VC calculations. Σ*n*_SF_/Σ*n*_GC_ = total sample size of all spaceflight (SF) groups/all GC groups. SF vs. GC: percentage difference between spaceflight and ground control. GC vs. VC: percentage difference between ground and vivarium control.

### Changes in trabecular bone turnover during spaceflight

**Fig. 5:**
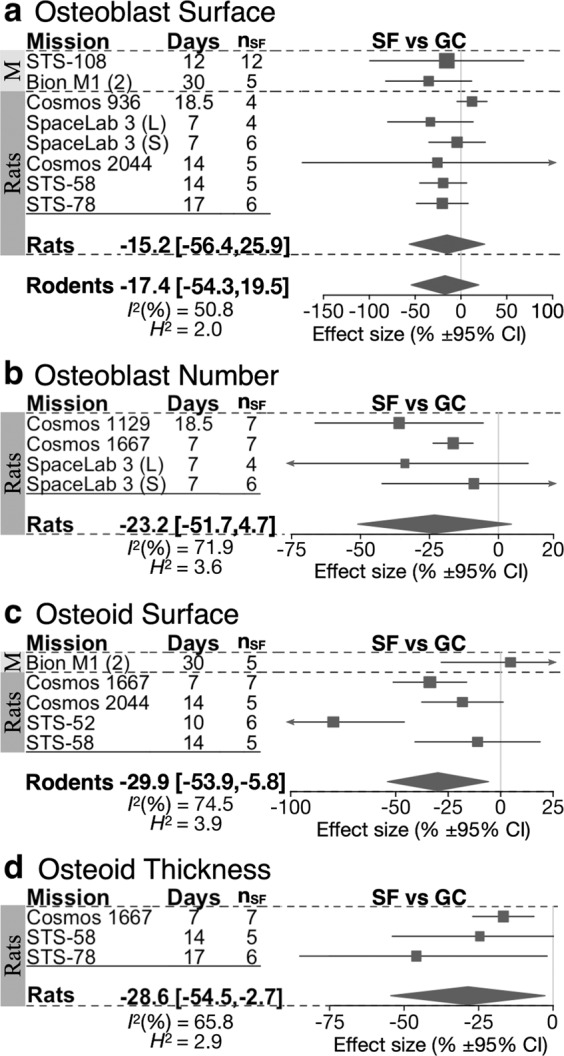
Forest plot of spaceflight induced changes in trabecular bone turnover parameters. Changes in osteoblast surface (**a**), osteoblast number (**b**), osteoid surface (**c**), and osteoid thickness (**d**) of space flight animals (SF) compared to ground control animals (GC). For each indicated species, missions are sorted by mission year (old to new); duration of spaceflight (Days), and the number of spaceflight animals (*n*_SF_) are indicated. Square/line: effect size (%) and 95% CI, the size of the square is proportional to *n*_SF_. Overall effect size (%) and 95% CI are indicated by diamonds for rats and rodents, *I*^*2*^, and *H*^2^ are given for rodents.

We next examined if spaceflight-induced bone deficits are associated with abnormal function of osteoblasts or osteoclasts. Osteoid surface (OS) and thickness (O.Th) were significantly lower in rodents by −29.9% [−53.9, −5.8] in OS and −28.6 [−54.5, −2.7] in O.Th; Osteoblast surface (Ob.S) and osteoblast number (N.Ob) demonstrated a trend to decrease compared to GC (Fig. 5). Comparison of ground and vivarium controls was available for only two missions for all osteoblast parameters except for Ob.S, for which GC and VC were not significantly different (Supplementary Tables 10–13). Heterogeneity for osteoblast parameters was moderate to high *I*^*2*^ > 50%. The osteoblast parameters were from trabecular skeletal regions, except for missions Cosmos 936 (Ob.S) and Cosmos 1667 (OS and O.Th), in which the measurements were from endocortical surface of the tibia diaphysis and metaphysis, respectively, and excluding these data resulted in homogeneous datasets for Ob.S and O.Th (*I*^*2*^ = 0), but not for OS (*I*^*2*^ = 74.9%). When only osteoblast indices in trabecular bone are considered, spaceflight resulted in a statistically significant reduction in Ob.S of −20.1% [−35.0, −5.1], OS −30.4% [−55.1, −5.8] and O.Th −36.2% [−60.2, −12.2]. Thus, osteoblast formation and function in rodents were negatively affected by spaceflight.

**Fig. 6:**
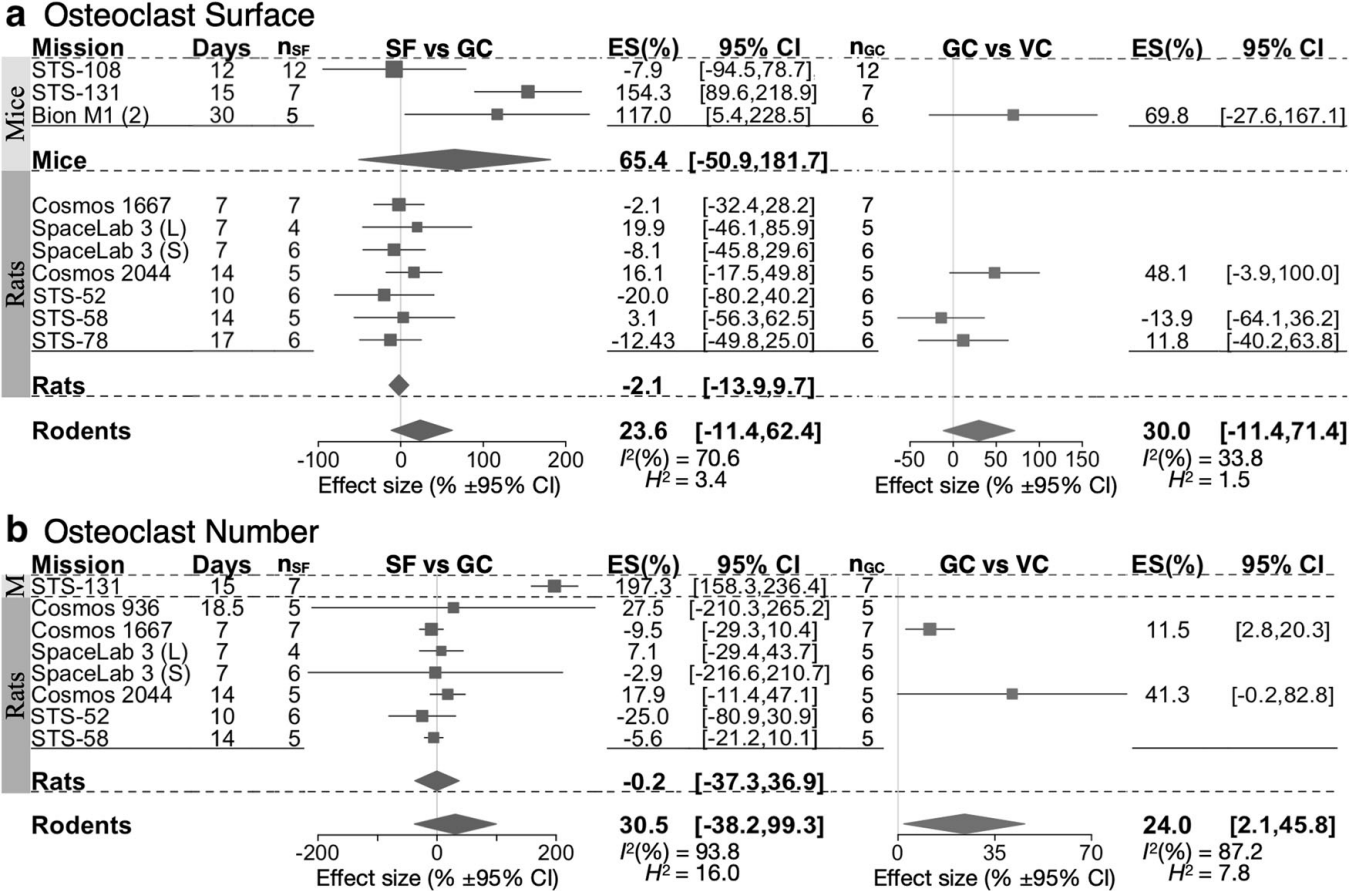
Forest plot of spaceflight induced changes to osteoclast parameters. Changes in osteoclast area (**a**), and osteoclast number (**b**) of space flight animals (SF) compared to ground control animals (GC) (Left); and GC compared to vivarium control animals (VC) (Right). For each indicated species, missions are sorted by mission year (old to new); duration of spaceflight (Days), and the number of spaceflight animals (*n*_SF_) are indicated. Square/line: effect size (%) and 95% CI, the size of the square is proportional to *n*_SF_. Overall effect size (%) and 95% CI are indicated by diamonds for mice, rats, and rodents, *I*^*2*^, and *H*^2^ are given for rodents.

In contrast to osteoblast parameters, changes in osteoclasts were inconsistent. Osteoclast surface (Oc.S) in SF mice demonstrated large study level increases in 2 of 3 datasets, however, it was unaffected in SF rats (Fig. 6a left). Osteoclast number (N.Oc) was higher in the one group of SF mice where it was measured, and was not significantly affected in spaceflight rats (Fig. 6b left). Moreover, comparing ground and vivarium controls demonstrated strong (10–70%) tendencies for study level increases (Fig. 6 right). Although the overall effect size for GC vs. VC comparisons only reached statistical significance for Oc.N, these data suggest that in rodents osteoclasts may be affected by spaceflight conditions other than microgravity. Heterogeneity was high for Oc.S and N.Oc datasets. The osteoclast parameters were from trabecular skeletal regions, except for missions Cosmos 936 (N.Oc) and one of the bones for mission Bion M1 (Oc.S); excluding these data did not significantly change the outcome. Thus, osteoclast parameters were unaffected in rats and variably affected in mice.

### Change in cortical bone parameters during spaceflight

**Fig. 7:**
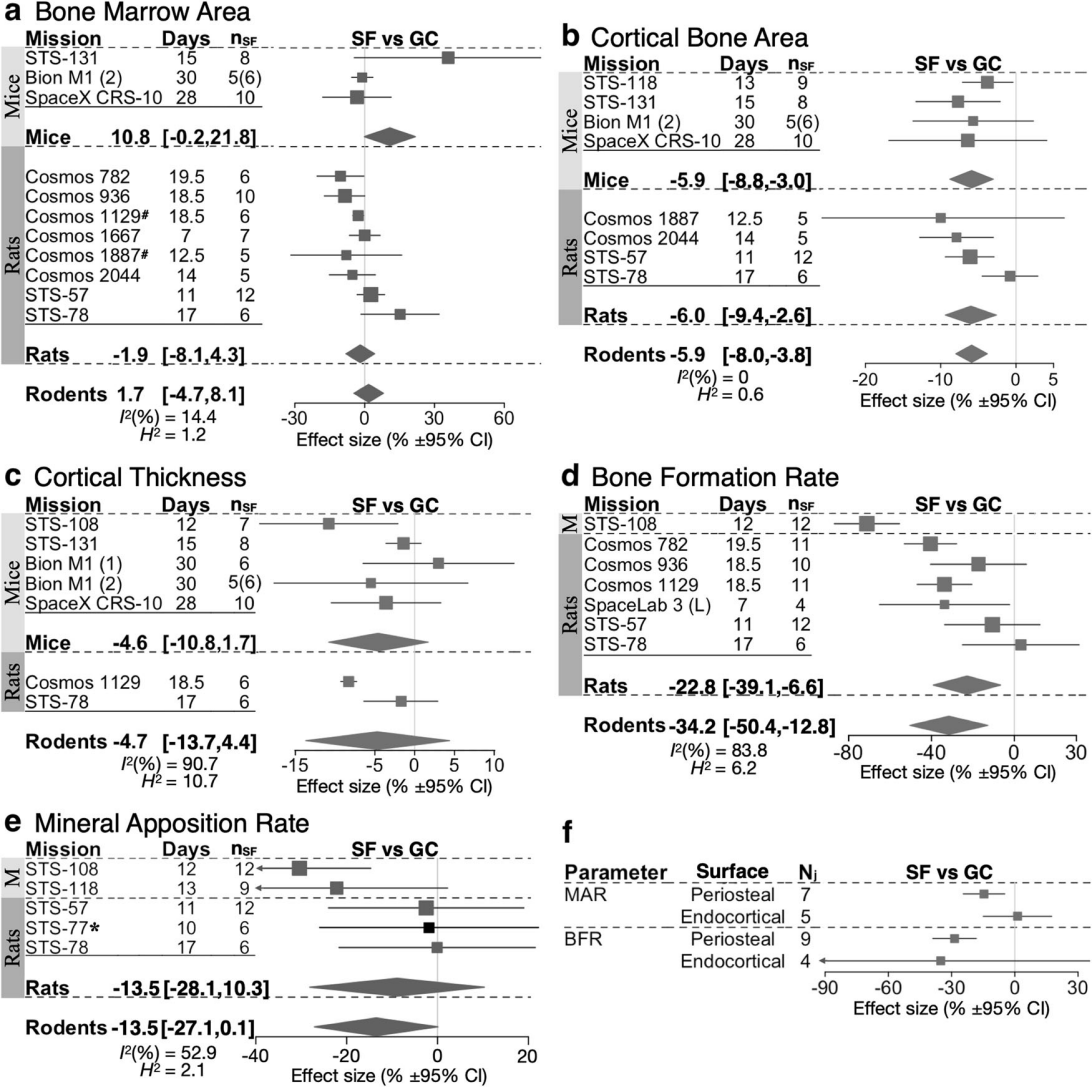
Forest plot of spaceflight induced changes to cortical bone parameters. **a**–**e** Changes in bone marrow area (**a**), cortical bone area (**b**), cortical thickness (**c**), as well as bone formation rate (**d**) and mineral apposition rate (**e**) for the diaphyses of long bones, of space flight animals (SF) compared to ground control animals (GC). For each indicated species, missions are sorted by mission year (old to new); duration of spaceflight (Days), and the number of spaceflight animals (*n*_SF_) are indicated. Square/line: effect size (%) and 95% CI, the size of the square is proportional to *n*_SF_. Overall effect size (%) and 95% CI are indicated by diamonds for mice, rats, and rodents, *I*^*2*^, and *H*^2^ are given for rodents. *missions where SF was compared to VC. #mission where Ma.Ar was derived from average marrow diameter (Av.Ma.Dm) as Ma.Ar = π(Av.Ma.Dm/2)^2^. **f** Change in BFR and MAR on the periosteal and endocortical surface of long bones in SF compared to GC. The number of measurements (*N*_*j*_) is indicated. Square/line: overall effect size (%) and 95% CI.

Cortical parameters analyzed were bone marrow area (Ma.Ar, which included data on bone marrow diameter (Ma.Dm) transformed as *π*(d/2)^2^), cortical area (Ct.Ar), and thickness (Ct.Th). Ma.Ar and Ct.Th did not significantly differ between SF and GC in mice and rats, while Ct.Ar was significantly lower in spaceflight mice and rats (Fig. 7a–c). GC did not significantly differ from VC for any cortical parameter (Supplementary Tables 14–16). The heterogeneity for cortical parameters was low, *I*^*2*^ < 15%, except for Ct.Th which showed high heterogeneity, *I*^*2*^ = 90.7%. The datasets of Ma.Ar, Ct.Ar, and Ct.Th are composed of measures taken in the diaphysis of long bones except for missions STS-131 (femoral neck), Bion M1 (animal group 1, ankle bones, and calcaneus), and SpaceX CRS-10 (rib). Removing these biological outliers did not change effect size and resulted in a homogeneous dataset for Ma.Ar with *I*^*2*^ = 0%. Cortical thickness measured in 4 primates was not significantly affected by spaceflight (Table 3). Thus, spaceflight resulted in cortical bone deficits, however, it was affected to a smaller degree compared to trabecular bone.

### Change in cortical bone turnover during spaceflight

Only measures of bone formation rate (BFR) and mineral apposition rate (MAR) from the cortical bone surface in the diaphysis of long bones were included in the analysis. This resulted in the exclusion of measures of MAR and BFR in the pelvis and thoracic vertebrae from STS-78^31^, and in the humeral metaphysis from STS-52 and non-included mission STS-41^50^. Both BFR and MAR were lower in spaceflight rodents by −34.2% [−50.2, −12.8] and −13.5% [−27.1, 0.1], respectively (Fig. 7d, e). There were no differences between GC and VC for BFR nor MAR (Supplementary Tables 17, 18). Heterogeneity was moderate to high for these parameters, *I*^*2*^ > 52%. When long bone measurements of MAR and BFR taken on the periosteal and endocortical surfaces were separated, we found that the reductions in MAR and BFR were only significant on the periosteal surfaces (Fig. 7f). Thus, bone formation on periosteal surfaces of cortical bone appears to be more affected by microgravity.

### Effects of covariates on spaceflight related changes in animal bone health

**Fig. 8:**
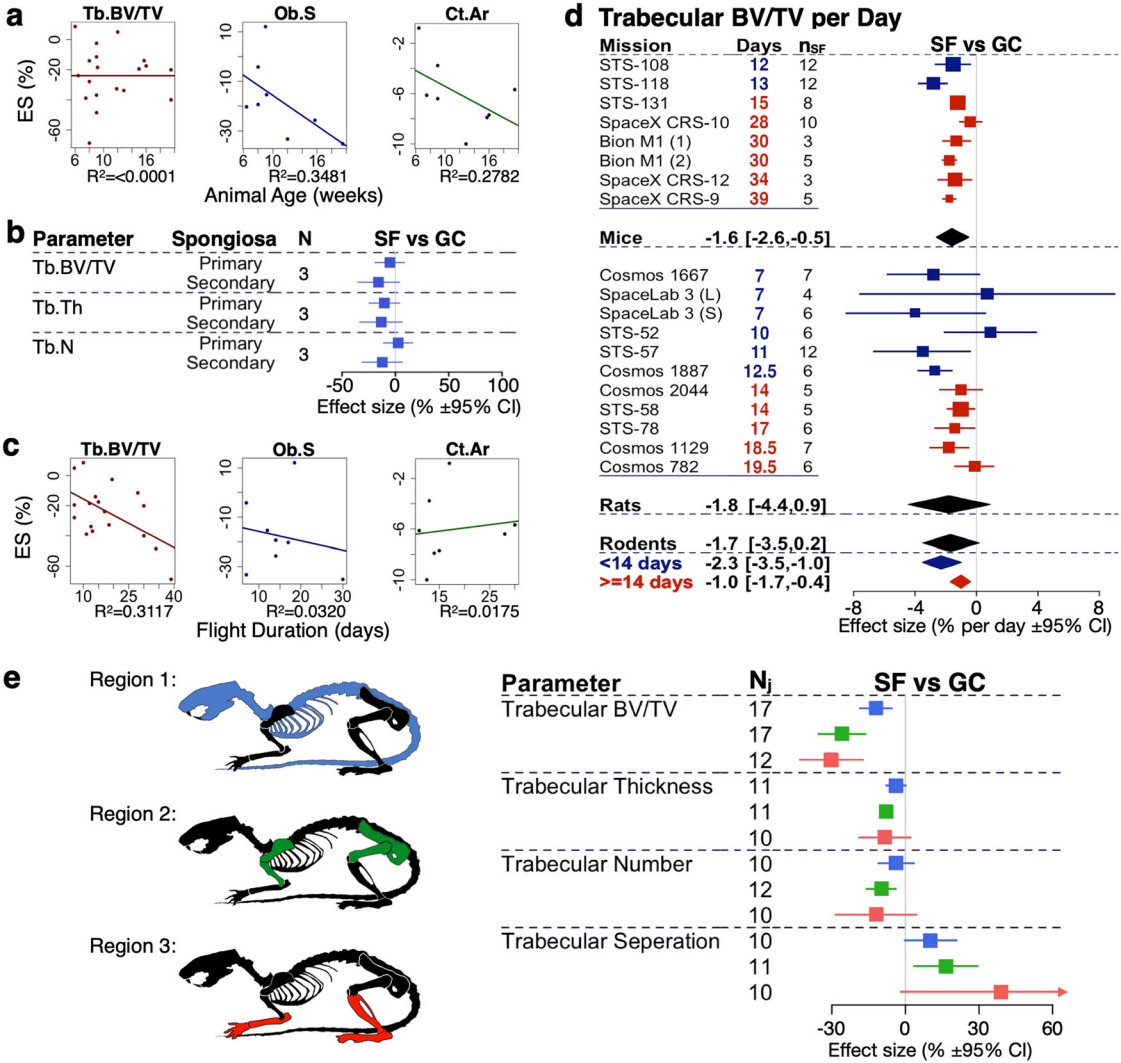
Exploratory analysis for the effect of covariates on spaceflight-induced changes in bone parameters. **a** Meta-regression of the Tb.BV/TV, Ob.S, and Ct.Ar as a function of animal age. **b** Subgroup analysis for Tb.BV/TV, Tb.Th, and Tb.N outcomes for primary and secondary spongiosa. **c** Meta-regression of the Tb.BV/TV, Ob.S, and Ct.Ar as a function of flight duration. **d** Forest plot of the rate of spaceflight induced change to Tb.BV/TV. **e** Subgroup analysis for Tb.BV/TV, Tb.Th, Tb.N, and Tb.Sp outcomes reported for individual rodent bones from region 1 (skull, vertebra, and thorax, blue), region 2 (pelvis, humerus, and femur, green), or region 3 (tibia and ankle bones, red) as illustrated on the left. For a and c, *R*^2^ is shown. For (**b** to **e**), *N* = number of missions, *n*_SF_ = spaceflight animal sample size, and *N*_*j*_ = number of measurements. Square/line: overall effect size (%) and 95% CI. For (**d**), in each indicated species, missions are sorted by duration (shortest to longest); duration of spaceflight (Days), and the number of spaceflight animals (*n*_SF_) are indicated. Square/line: effect size (%) and 95% CI; dark blue: missions less than 14 days; dark red: missions 14 days or longer. Overall effect size (%) and 95% CI are indicated by diamonds for mice, rats, and rodents (black), rodents on short duration (dark blue), and long duration (dark red) missions.

We next examined the contribution of covariates to the overall outcomes using sub-group analysis and meta-regression. First, we examine if animal characteristics, such as age, sex, and strain affect the overall outcome. Using linear regression analysis, we have found that rodent age was weakly associated with changes in osteoblast surface and cortical area, but not with Tb.BV/TV (Fig. 8a). Subgroup analysis further demonstrated that in animals 10 weeks of age or older, larger changes were observed in Tb.N, Ob.S, and Oc.S, while Ct.Th was more affected in younger animals (Supplementary Fig. 3a). When we compared trabecular parameters in instances when both primary and secondary spongiosa of a single bone were analyzed, we observed that changes in Tb.BV/TV, Tb.N, and Tb.Th in secondary spongiosa was greater than in primary spongiosa (Fig. 8b). Animal sex or strain did not significantly affect the outcome (Supplementary Fig. 3b, c).

Next, we examined if mission-related differences affected the outcome. Spaceflight duration did not significantly correlate with changes in Ob.S and Ct.Ar but was weakly associated with changes in Tb.BV/TV when assessed using meta-regression analysis (Fig. 8c). Moreover, subgroup analysis by mission durations shorter or longer than 2 weeks, demonstrated no significant difference for any parameters (Supplementary Fig. 4a). To estimate the rate of accumulation of bone deficits in space, we divided individual outcomes of our largest parameter dataset, Tb.BV/TV, by the mission duration. Although not statistically significant, the deficits in Tb.BV/TV per day was smaller in long spaceflights than in short spaceflights (Fig. 8d). We estimated the rate of accumulation of trabecular bone deficits as −1.7%/day [−3.5, 0.2], or −1.0%/day [−1.7, −0.4] when taking into account only long-duration missions. We also assessed if individual vs. group housing affects the outcomes, however, no differences were found except for Tb.N, which changed significantly greater when animals were housed individually (Supplementary Fig. 4b). Comparing outcomes by the space agency, we determined no significant difference between space agencies (Supplementary Fig. 4c).

Study-related differences included measurement techniques, presence of sham operation, sacrifice delay, and ground control conditions. For all trabecular and cortical architectural parameters, the division of measurement technique (Histology vs. μCT) coincided with the species difference of rats and mice preventing us from conducting any further meaningful subgroup analysis. In sham-operated rodents Tb.Th was affected significantly less than in naïve animals (Supplementary Fig. 5a). The sacrifice delay did not significantly affect the outcomes in subgroup analysis, although the change in Ob.S was associated with prolonged sacrifice delay in meta-regression analysis (Supplementary Figs. 5b, 6). When ground control groups were divided by the degree to which they mimic the environmental conditions of spaceflight other than the microgravity, we observed no association between the fidelity of the GC and spaceflight-induced changes, suggesting that they were primarily driven by microgravity (Supplementary Fig. 6c).

In astronauts, bone loss is strongly affected by its position in relation to the gravitational vector^5,74^. To assess if a similar trend is present in rodents, we performed a sub-group analysis of bones from different regions: region 1 that included calvaria, vertebrae, ribs, and sternum; region 2 with pelvis, humerus, and femur; and region 3 with tibia and ankle bones (Fig. 8e left). Changes in trabecular parameters were larger in bones located more distal from the axial skeleton (Fig. 8e right), however, the mean effect sizes were not significantly different between the regions. Among other parameters, Ct.Th, Ob.N, OS, and BFR demonstrated significant changes only in regions 2 and/or 3, while changes in Ob.S were only significant in regions 1 and 2 (Supplementary Fig. 7). These data suggest that bone position in relation to the gravitational vector may be important for rodents, however, targeted studies investigating these relationships would be required.

## Discussion

We systematically reviewed and quantitatively synthesized the literature on bone health in space-faring rodents and primates. We report that bone mass is lower in spaceflight rodents and primates, with indications that microgravity is the driving factor inducing bone deficits. Deficits in trabecular bone were larger than in cortical bone and subgroup analysis suggested that distal skeleton was affected more than axial. Osteoblast indices in rodent trabecular bone were significantly lower, however, osteoclast numbers were not affected in rats, and were variably affected in mice. Even though the degree of bone deficit was found to poorly correlate with mission duration, the rate of accumulation of trabecular bone deficit was estimated as −1.7% [−3.5, 0.2] per day, which is much higher than the estimates of bone loss available for humans. Taken together, our data indicate that microgravity induces bone deficits in rodents and primates, and the data suggest that the prevalent mechanism is suppression of bone formation.

We have found that during the 4–39 days space missions rodents accumulated a deficit of −24.1% [−43.4, −4.9] in trabecular bone tissue, which translates to the rate of 1.7% of trabecular bone deficit per day. In the much smaller dataset for primates, the bone deficit after 11.5–14 day missions was equally high, −25.2% [−35.6, −14.7] or 1.9% per day. These estimates for trabecular bone deficits in spaceflight rodents and primates are much greater than estimates of bone loss for astronauts which have been reported as 0.7–2.7% per month^4,5,75,76^. Nevertheless, similar deficits of 15–50% in tibial and femoral trabecular bone volume were reported in 2–4 week-long hindlimb unloading studies in rats and mice^77–81^, which can be recalculated to 1.1–3.5% per day. We observed that no single parameter was strongly associated with mission duration. In astronauts, changes to bone were also highly variable for missions less than 30 days in duration^5^. Of spaceflights studying bone in rodents, only 3 missions were longer than 30 days, one of which (Mice Drawer System (MDS)) was excluded since of the 3 wild type mice aboard, only 1 returned to Earth alive^70^, preventing us from extracting meaningful quantitative data from it. Thus, continuous measurements of bone parameters in longer missions (>30 days) are required to determine the dynamic association between the duration of exposure to microgravity and bone health.

We have identified several instances of notable regional differences in bone response to microgravity. First, we have found that the deficits in trabecular bone were much greater than those in cortical bone in space-traveling rodents. Similarly, higher deficits in trabecular bone compared to cortical were reported in studies of hindlimb unloaded rats^82^, as well as in astronauts^75^. In cortical bone, bone formation was only significantly suppressed on the periosteal surface, which is supported in the observation that Ct.Ar, but not Ma.Ar, was significantly lower in spaceflight rodents. Similar changes in cortical bone formation were observed in hindlimb unloading studies in mice^82^. Within trabecular bone, we found that rodents exhibited relatively greater deficits in secondary spongiosa compared to primary spongiosa. Secondary spongiosa was also found to be more affected compared to primary in rats after hindlimb unloading^61,83,84^. However, in the model of immobilization due to sciatic denervation, the bone loss was isolated to primary spongiosa^85^. Of interest, we also observed a weak association of osteoblast suppression and cortical bone loss with older age in space-traveling rodents. In contrast, extensive and well-controlled studies of the impact of age on bone health in hindlimb unloaded rats reported the opposite trend—higher bone deficits in younger animals^77,80^. In this regard, it is important to note that the oldest spaceflight rodents were relatively young, 20 weeks of age at the start of the mission, and therefore more studies are needed to fully understand the impact of age on bone health in space. Similarly, even though dramatic sex-related differences were reported in hindlimb unloaded rats^81^, the effect of sex was poorly investigated in spaceflight animals, with no data available for female rats or primates, and only some mouse studies reporting changes in females.

In humans, the significant association between bone loss and the bone position relative to the gravitational vector was identified^5,74^. Although it is more difficult to account for an equivalent gravitational vector in rodents, we attempted to assess the regional difference in bones of rodents assuming their quadrupedal movement. We have found that similar to humans, in rodents, distal skeletal regions exhibited a trend of increased trabecular bone deficits compared to axial skeletal regions. Furthermore, in two mouse studies that measured total BV/TV of the calvariae^36,40^, an increase was reported. These data suggest that local factors, including microgravity-induced redistribution of body fluid^86^, or change in mechanical environment^87^ likely contribute to poor bone health.

We demonstrate that spaceflight is associated with strong inhibition of bone formation in rats, mice, and primates, while osteoclast indices were not affected in rats, variably affected in mice, and not reported in primates. In contrast, in astronauts, resorption was found to rise rapidly, reaching a sustained 2-fold increase for the duration of the spaceflight, while formation was decreased or unchanged at the beginning of the mission after which it gradually increased over time^5^. However, the direct comparison between animal and human data is difficult due to important methodological differences in data acquisition. While in animals bone turnover is predominantly assessed histologically at the end of the space mission, in humans, biochemical markers of bone formation and resorption are measured in serum or urine, allowing for assessment during the spaceflight mission. Importantly, most histological markers only indicate the change in bone cell numbers, while circulating markers reflect both changes in the number and function of bone cells. Nevertheless, we believe that the data conclusively indicate that bone formation is inhibited in animals during spaceflight, because indices related to osteoblast numbers (osteoblast numbers and surface), and histomorphometric measures of osteoblast function (osteoid surface and thickness, mineral apposition rate, and bone formation rate), were lower in spaceflight rodents or primates. In contrast, bone resorption data for spaceflight animals is less consistent and more difficult to compare to humans. Osteoclast numbers or surfaces uniformly did not change in rats, while in mice missions STS-131 and Bion M1 reported strong increases in osteoclast number and surface, but mission STS-108 demonstrated no change. Osteoclast function was assessed using circulating markers in two missions: in mission STS-108, that reported no change in osteoclast number, circulating TRAP5b was higher^33^; and in mission STS-118, a 13 days mission with mice for which no histological osteoclast data is available, circulating TRAP5b did not change^34^. Thus, although the data suggest that there may be a difference in the response of bone cells to microgravity between rodents and humans, and/or between mice and rats, we are limited by different nature of measurements in animals and humans, and small sample size for mice. Therefore, more experiments assessing both bone cell numbers and function, especially for osteoclasts, are required to understand the spaceflight-induced changes in bone turnover.

This study has attempted to quantitatively integrate nearly 50 years of bone research in spacefaring animals. The limitations of this analysis included *i)* the differences in the design of experiments in individual missions, *ii)* inconsistent reporting, and *iii)* the need to meta-analytically combine data performed using different protocols over a large time interval. Experimental design of individual missions evolved with time, however notably, there was little data from spaceflights longer than 30 days, and there were no inflight measures of bone turnover or quality, which prevented us from assessing the long-term and dynamic effects of microgravity on the animal bone. Of specific importance for animal experiments, is the design of the ground control, which aimed to model the parameters of spaceflight other than microgravity, which was vastly different between missions. While this resulted in a limitation of comparing experimental groups to very different controls, it also allowed us to perform a preliminary assessment of the relative effects of stressors associated with spaceflight other than microgravity. Since the extent of modeling the stressors in ground control groups was not associated with differential bone deficits, we concluded that microgravity is the main driver of these changes. The most rigorous control for the specific effects of microgravity was in-space artificial gravity, which was performed during three missions, Cosmos 936, SpaceX CRS-9, and CRS-12. When the in-flight 1 g group was used as a “ground control”, the effect sizes for bone changes were not smaller than in missions with ground controls of lower fidelity. In addition, for Cosmos 936 which also had an associated vivarium control group, ground to vivarium control effect sizes and 95% CI were not significantly different from other ground to vivarium control comparisons, altogether suggesting that microgravity is the driving factor for bone loss in space. Nevertheless, we did identify several parameters, including trabecular thickness, and osteoclast surface, and number that appears to be specifically affected in ground control compared to vivarium control groups, suggesting that other spaceflight-associated factors may contribute to those changes. The second set of limitations was relevant to data reporting in the manuscripts. In multiple instances, inconsistent reporting of animal treatment between papers reporting the same mission was observed. Rodent death was not uncommon during spaceflight, however, it was infrequently reported, even though it reflects the stressful conditions during a particular mission, which then could not be accounted for in our analysis. Specifications regarding bone surfaces analyzed in addition to control and spaceflight animal treatment/housing were often vague making categorizing for subgroup analysis difficult. In addition, the degree of movement, which has the potential to affect bone health^4^, was never reported in the included articles in rodents. This represents a significant shortcoming in reporting of the outcomes of animal experiments in space, since for several missions animal behavior data has been collected^88^. Therefore, similar to human studies^89^, improving reporting practices of animal experiments by the Space Life Sciences Programs is critically important. The third set of limitations was related to performing a meta-analysis on studies completed over a considerable interval of time with vastly different protocols. This resulted in our dataset being moderate to highly heterogeneous for 15 out of the 17 parameters. While we attempted to identify all possible factors that may account for the high degree of heterogeneity, no single factor accounted for a major amount of variation in any of the measured outcomes. Since our analysis indicates low publication bias, high heterogeneity likely reflects the multifactorial nature of microgravity-induced bone changes, which can only be investigated through the analysis of larger datasets.

In conclusion, we demonstrate that meta-analysis of animal spaceflight data provides important additional information regarding the effect of microgravity on animal physiology, in particular allowing to perform comparative studies, which otherwise are financially and technologically challenging. Our studies on animals and humans^5^ demonstrate that microgravity-induced deterioration of bone health is a complex phenomenon, with strong regional and temporal differences, as well as potentially different mechanisms of adaptation in different species. In the future, longer missions with planned in-flight data collection are needed to understand the dynamics of changes in bone tissue and especially bone turnover, which appears to be different between humans and rodents. For nonhuman animals, in particular, it is also important to relate the changes in bone to the movement patterns and activity, which are rarely provided in bone health-focused studies. The quantitative estimates of spaceflight-related changes in bone health provided by our study will inform future studies and help in determining the underlying mechanisms of observed effects.

## Methods

This study was compliant with the Preferred Reporting Items for Systematic Reviews and Meta-analysis (PRISMA) statement. Refer to Supplementary Table 1 for PRISMA Checklist.

### Search strategy, inclusion criteria, and quality assessment

A systemic search strategy using terms related to bone, space travel, and animals, including the names of individual missions, bones, and species of nonhuman vertebrates (Supplementary note 1) was constructed by a medical librarian (MM). Medline, Embase, PubMed, BIOSIS Previews, and Web of Science were searched on November 2nd, 2017. An updated search was performed on November 1st, 2019. In addition, a manual search of the NASA Technical Report Server and articles referenced the compendium of animal and cell spaceflight experiments compiled by Ronca et al.^9^ was performed. Studies in any language were considered. Title and abstract screening for the original search was performed independently by S.D.C. and S.F.C., and for the update by S.V.K. The inclusion criteria were that the article describes any vertebrate species that was taken on a space mission. Studies describing invertebrate animals, humans, or Earth-based spaceflight simulations were excluded. After intermediate analysis, only studies describing spaceflight results for mice, rats, and primates were included in full-text screening for quantitative measurements related to bone health, which was performed by S.D.C., S.F.C., and M.G. for the initial search and by S.V.K. and M.G. for the update. In the final meta-analysis, we included the studies that presented quantitative measurements of trabecular and cortical architecture or bone turnover for bones of axial and appendicular skeleton excluding facial bones. Animals that were pregnant, or received surgery other than a sham, abnormal diet, or hormone supplements, were excluded. Papers presenting average data without a measure of variation were excluded. Included papers were scored for reporting quality (Supplementary note 2), if two different species were reported in a single paper, they were scored independently.

### Data extraction

For studies included after abstract/title screening, the year of publication, animal species, and physiological system studied were recorded. For studies that were included in a meta-analysis the following data were independently extracted by M.G. and S.F.C. and verified by J.F.: name and duration of the mission, animal species; animal sample size (*n*) of spaceflight, ground control, vivarium control, and delayed simulation (when applicable); bone and bone region being measured; and mean, the median and median percent difference in the 18 bone health parameters (Table 1); standard deviations, standard errors of the mean and/or interquartile ranges; day or range of days when measurements were performed. If the type of measure of the dispersion was not stated, it was assumed to be a standard error, which ensures a conservative estimate. If a range of sample sizes was reported, the smallest value was extracted. Extracted study characteristics for covariate analysis included: animal strain, age, sex, spaceflight group sacrifice delays, single vs. grouped spaceflight habitat, the space agency, treatment conditions of the ground control group, and the presence of sham operations. The information regarding a specific mission was pooled from all applicable articles. When different data for apparently identical samples were presented in two papers, we included the data from the study with the higher quality score. For spaceflight group sacrifice delay, if a range of time was given, the largest time interval was used. Alternate terms used for included parameters are presented in Supplementary Table 2.

### Measurement-level outcomes

Three types of the control group were used: the vivarium control (VC), where animals lived in a standard laboratory habitat; the ground control (GC), where some or all aspects of space flight excluding microgravity were modeled; the delayed simulation (DS), only seen in primate studies, where spaceflight animals were placed in an earth-based GC habitat several weeks following recovery. When available, we used GC as the comparison group. If multiple GC groups were used, we treat the group that most closely matched flight conditions as the GC. When GC was not available, we used VC or DS as the comparison group. For each individual measurement *j*, we extracted the mean space flight (SF) values, *μ*_SF*j*_, and the mean comparison control (CC) values, *μ*_CC*j*_ with the corresponding standard errors se_*j*_, or standard deviations sd_*j*._ If sd_*j*_ was extracted, it was converted to se_*j*_ by dividing by the square root of sample size *n* of the corresponding group, such as *n*_SF_ for spaceflight and *n*_CC_ for comparison control. When median *P* and interquartile range *x*_upper _− *x*_lower_ were given, *μ*_*j*_ was calculated as *μ*_*j*_ = (*x*_upper_ + *P* + *x*_lower_) with: ${\mathrm{se}}_j = x_{{\mathrm{upper}}} - x_{{\mathrm{lower}}}/\sqrt n \times 2.7$. For each measurement, we calculated the percentage difference, *θ*_*j*_, between *μ*_SF*j*_ and *μ*_CC*j*_ using Eq. (1).

1 $$\theta _j = \frac{{\mu _{{\mathrm{SF}}_j} - \mu _{{\mathrm{CC}}_j}}}{{\mu _{{\mathrm{CC}}_j}}} \times 100{\mathrm{\% }}$$

Normalized standard errors SE_*j*_ were calculated as SE_*j*_ = se_*j*_/*μ*_cc*j*_. The standard deviation for percentage difference of a single measurement *σ*_*j*_ was calculated assuming that the SF and CC groups were independent using Eq. (2).

2 $$\sigma _j = \sqrt {{\mathrm{SE}}_{{\mathrm{SF}}_j}^2 + {\mathrm{SE}}_{{\mathrm{CC}}_j}^2} \times 100{\mathrm{\% }}$$

### Mission-level outcomes

Data for multiple *b* bones or bone regions presented in one or more studies for the same group of animals were pooled as unweighted averages $\theta _i = \frac{{{\sum} {\theta _j} }}{b}$ to represent the outcome or effect size of a single mission *i*. In two instances (Bion M1 and SpaceLab 3) where the data for two animal groups on the same mission were reported separately, they were treated as two independent missions. Equation (3) was used to calculate the overall standard error for each mission.

3 $${\mathrm{SE}}\left( {\theta _i} \right) = \sqrt {\frac{{{\sum} {\sigma _j^2} }}{{{\sum} {\left( {n_{{\mathrm{SF}}} + n_{{\mathrm{CC}}}} \right)} }}}$$

### Meta-analytic model and global outcome

Since the mission-level data encompass outcomes from many spaceflights performed over a long period of time in multiple animal species, we rejected the fixed-effect model in favor of the random-effects model. However, since individual sample sizes were small (between 4 and 12), the variance is not a representative measure of the better estimate of the mean, making the variance-based weighting scheme biased. Therefore, to calculate the global effect size ${\it{\widehat {\uptheta}}}$, the mission-level outcomes *θ*_*j*_ were weighted by the sample size of spaceflight animals *n*_SF_ using Eq. (4).

4 $${\it{\widehat {\uptheta}}} = \frac{{\mathop {\sum}\nolimits_i {\theta _i \times n_{{\mathrm{SF}}}} }}{{\mathop {\sum}\nolimits_i {n_{{\mathrm{SF}}}} }}$$

When combining data from multiple articles with differing sample size *n*_SF_, the smallest sample size among them was used for global outcome calculations. Global outcomes were calculated for mice, rats, primates, and rodents overall.

To account for heterogeneity between the studies, we adapted the approach developed by Standley and Doucouliagos^90^, in which we adjusted the pooled standard error by the factor representing the degree of heterogeneity within the dataset. We calculated the adjusted heterogeneity estimator *H*^*2*^ to represent the variability of *θ*_*i*_ from the global outcome ${\it{\widehat {\uptheta}}}$ within *N* mission-level outcomes as follows using Eq. (5).

5 $$H^2 = \frac{{\mathop {\sum}\nolimits_i {\left( {\frac{{\theta _i}}{{{\mathrm{SE}}\left( {\theta _i} \right)}} - \frac{{{\it{\widehat {\uptheta}}}}}{{{\mathrm{SE}}\left( {\theta _i} \right)}}} \right)} ^2}}{{\left( {N - 1} \right)}}$$

Equation (6) was used to calculate the standard error of the global outcome ${\it{\widehat {\uptheta}}}$.

6 $${\mathrm{SE}}\left( {\it{{\widehat {\uptheta}}}} \right) = \sqrt {\frac{{H^2}}{N}} \times \root {2} \of {{\frac{{\mathop {\sum}\nolimits_i {\left( {{\mathrm{SE}}\left( {\theta _i} \right)^2 \cdot \left( {n_{{\mathrm{SF}}} - 1} \right)} \right)} }}{{\mathop {\sum}\nolimits_i {\left( {n_{{\mathrm{SF}}} - 1} \right)} }}}}$$

This meta-analytic model provides the unbiased estimate of the central tendency and conservative estimates for the 95% confidence intervals (CI) which was determined as 95% CI${\mathrm{CI}} = {\it{\widehat {\uptheta}}} \pm {\mathrm{z}}_{\left( {1 - {\upalpha}/2} \right)} \times {\mathrm{SE}}( {\it{{\widehat {\uptheta}}}}) = {\it{\widehat {\uptheta}}} \pm 1.96 \times {\mathrm{SE}}( {\it{{\widehat {\uptheta}}}})$. To assess the influence of spaceflight associated conditions other than microgravity, we similarly calculated the percentage difference of GC from VC.

### Rate of change

To estimate the rate of change per day, we used mission-level outcomes from the parameter with the largest dataset, trabecular BV/TV. For each mission, the percentage difference in trabecular BV/TV was divided by the duration of each mission *Days* to calculate $\theta _{i\,{\mathrm{per}}\,{\mathrm{day}}} = \frac{{\theta _i}}{{{\mathrm{Days}}}}$ and ${\mathrm{se}}\left( {\theta _i} \right)_{{\mathrm{per}}\,{\mathrm{day}}} = \frac{{{\mathrm{se}}\left( {\theta _i} \right)}}{{{\mathrm{Days}}}}$, which were then used in the meta-analytic model. Although it is unlikely that changes in bone mass in space occur linearly, with only 2 measurements for each group, any rate estimate other than linear would inevitably result in over-fitting.

### Heterogeneity and publication bias analysis

To quantify heterogeneity, we calculated *H*^*2*^ as described above and *I*^2^ as $I^2 = \frac{{H^2 - 1}}{{H^2}}$. To examine the contribution of individual datasets we used single data exclusion analysis when one mission-level outcome was excluded and its effect on heterogeneity on the remaining dataset was calculated; and cumulative data exclusion analysis when multiple mission-level outcomes were excluded in the order of their contributing heterogeneity. To assess publication bias, a funnel plot was used to plot the distribution of the standard errors relative to estimated mission-level outcomes. All the studies were included in the final analysis independent of their contribution to heterogeneity or potential bias.

### Additional analysis

We performed subgroup analysis on 11 characteristics: age of animals, the strain of rats, sex of mice, flight duration, individual vs. grouped housing conditions, the space agency, the conditions of ground control, the delay time of SF animal sacrifice, presence of sham operation, the quality score of papers and skeletal region of measurements. For strain, sex, the space agency, ground control, and housing condition, the subgroup analysis was performed by a categorical value for each mission using the mission-level effect size and 95% CI as described above. For continuous values of age of animals, duration of flights, sacrifice delay, and quality score, the missions were divided into 2 groups of approximately equal size for sub-group analysis; or a linear regression against the continuous variable was performed for representative parameters for trabecular and cortical structure and turnover. For the quality score, measurement-level outcomes from a single article were combined to create a paper-level outcome, *θ*_*p*_ and associated measure of variance SE(*θ*_*p*_), replacing mission-level outcomes in subgroup analysis and linear regression. For the skeletal region, measurement-level outcomes were combined. For quality score and bone region analysis, the global effect size ${\it{\widehat {\uptheta}}}$ and standard error ${\mathrm{SE}}( {\it{{\widehat {\uptheta}}}} )$, were estimated using the random-effects model with the Hedges estimator *τ* for unit weight $w_i = \frac{1}{{{\mathrm{SE}}\left( {\theta _i} \right)^2 + \tau ^2}}:{\it{\widehat {\uptheta}}} = \frac{{\mathop {\sum}\nolimits_i {\left( {\theta _i \cdot w_i} \right)} }}{{\mathop {\sum}\nolimits_i {\left( {w_i} \right)} }},\,{\mathrm{SE}}\left( {\it{{\widehat {\uptheta}}}} \right) = \frac{1}{{\root {2} \of {{\mathop {\sum }\nolimits_i \left( {w_i} \right)}}}}$^91^. Subgroup analysis was only performed on parameters with 6 or more mission-level, paper-level, or measurement-level outcomes.

### Outcome reporting

Data are presented as effect size or percentage difference between spaceflight and ground control animals or ground control and vivarium control with lower and upper limits of 95% CI as: ES(%) [lower CI, Upper CI].

### Software

Endnote X7 and Rayyan were used for the management of references. WebPlot digitizer was used in data extraction. Numbers (version 4.1.1) were used for data management. R (version 1.1.463) was used for meta-analysis and associated calculations. R (version 1.1.463), JASP (version 0.10), and MATLAB (MATLAB online) were used for initial figure preparation.

### Reporting summary

Further information on research design is available in the Nature Research Reporting Summary linked to this article.

## Supplementary information

Supplementary Information

Reporting Summary

## Data Availability

Raw data can be made available upon reasonable request to author Matthew Goldsmith (matthew.goldsmith2@mail.mcgill.com).

## Code availability

Code can be made available upon reasonable request to author Jingyan Fu (jingyan.fu@mail.mcgill.ca).

## References

[1] NASA. *Artemis Plan: NASA’s Lunar Exploration Program Overview*. 1–74 (2020).

[2] NASA. *NASA’s Journey to Mars*. https://www.nasa.gov/content/nasas-journey-to-mars (2014).

[3] Mars One. *Current Mission Status*. http://www.mars-one.com/about-mars-one/current-mission-status (2016).

[4] Vico L, Hargens A (2018). Skeletal changes during and after spaceflight. Nat. Rev. Rheumatol..

[5] Stavnichuk M, Mikolajewicz N, Corlett T, Morris M, Komarova SV (2020). A systematic review and meta-analysis of bone loss in space travelers. NPJ Microgr..

[6] Gray, T. *A Brief History of Animals in Space.* https://history.nasa.gov/animals.html (2014).

[7] Morey-Holton ER, Hill EL, Souza KA (2007). Animals and spaceflight: from survival to understanding. J. Musculoskelet. Neuronal Interact..

[8] Pielke R, Byerly R (2011). Shuttle programme lifetime cost. Nature.

[9] Ronca, A. E., Souza, K. A. & Mains, R. C. *Translational Cell and Animal Research in Space: Ames Research Center.* 1965-2011. (NASA Special Publication, 2015).

[10] Asling, C. W. *Histological Studies on Tibial Bone of Rats in the 1975 Cosmos-782 Flight: I. Endochondral Osteogenesis; Medullary Bone Turnover*. Report No. TM-78525, 276–290 (NASA, 1978).

[11] Morey ER, Baylink DJ (1978). Inhibition of bone formation during space flight. Science.

[12] Morey-Holton, E., Turner, R. T. & Baylink, D. J. *Quantitative Analysis of Selected Bone Parameters*. Report No. TM-78526, 135–178 (NASA, 1978).

[13] Judy, M. M. *Quantitative Analysis of Selected Bone Parameters: Supplement 3A: Trabecular Spacing and Orientation in the Long Bones*. Report No. TM-81289, 177–198 (NASA, 1981).

[14] Wronski, T. J. & Morey-Holton, E. *Quantitative Analysis of Selected Bone Parameters*. Report No. TM-81289, 101–125 (NASA, 1981).

[15] Jee WS, Wronski TJ, Morey ER, Kimmel DB (1983). Effects of spaceflight on trabecular bone in rats. Am. J. Physiol..

[16] Rogacheva IV, Stupakov GP, Volozhin AI, Pavlova MN, Poliakov AN (1984). [Characteristics of bone tissue of rats after flight aboard biosputnik Kosmos-1129]. Kosm Biol. Aviakosm Med..

[17] Kaplanskii AS, Durnova GN, Sakharova ZF, Il’ina-Kakueva EI (1987). [Histomorphometric analysis of the bones of rats on board the Kosmos 1667 biosatellite]. Kosm. Biol. Aviakosm Med..

[18] Vico L (1988). Trabecular bone remodeling after seven days of weightlessness exposure (BIOCOSMOS 1667). Am. J. Physiol..

[19] Wronski TJ, Morey-Holton ER, Doty SB, Maese AC, Walsh CC (1987). Histomorphometric analysis of rat skeleton following spaceflight. Am. J. Physiol..

[20] Doty S, Morey-Holton E, Durnova G, Kaplansky A (1990). Cosmos 1887: Morphology, histochemistry, and vasculature of the growing rat tibia. FASEB J..

[21] Vailas AC (1990). Effects of spaceflight on rat humerus geometry, biomechanics, and biochemistry. FASEB J..

[22] Zerath E (1990). Effets osseux de 13 jours de microgravité chez le rat. Résultats de BIOCOSMOS 1887. Trav. Sci..

[23] Vailas AC (1992). Adaptation of young adult rat cortical bone to 14 days of spaceflight. J. Appl. Physiol..

[24] Vico L, Bourrin S, Genty C, Palle S, Alexandre C (1993). Histomorphometric analyses of cancellous bone from COSMOS 2044 rats. J. Appl. Physiol..

[25] Turner RT, Evans GL, Wakley GK (1995). Spaceflight results in depressed cancellous bone formation in rat humeri. Aviat. Space Environ. Med..

[26] Westerlind KC, Turner RT (1995). The skeletal effects of spaceflight in growing rats: Tissue-specific alterations in mrna levels for TGF-β. J. Bone Miner. Res..

[27] Zerath E (1996). Effects of spaceflight and recovery on rat humeri and vertebrae: histological and cell culture studies. J. Appl. Physiol..

[28] Lafage-Proust MH (1998). Space-related bone mineral redistribution and lack of bone mass recovery after reambulation in young rats. Am. J. Physiol..

[29] Bateman TA (1998). Histomorphometric, physical, and mechanical effects of spaceflight and insulin-like growth factor-I on rat long bones. Bone.

[30] Wronski TJ (1998). Lack of effect of spaceflight on bone mass and bone formation in group-housed rats. J. Appl. Physiol..

[31] Zerath E (2000). Spaceflight inhibits bone formation independent of corticosteroid status in growing rats. J. Bone Miner. Res..

[32] Vajda EG, Wronski TJ, Halloran BP, Bachus KN, Miller SC (2001). Spaceflight alters bone mechanics and modeling drifts in growing rats. Aviat. Space Environ. Med..

[33] Lloyd SA (2015). Osteoprotegerin is an effective countermeasure for spaceflight-induced bone loss in mice. Bone.

[34] Ortega, A. M. et al. *ASME 2013 Summer Bioengineering Conference Vol. 1A: Abdominal Aortic Aneurysms; Active and Reactive Soft Matter; Atherosclerosis; BioFluid Mechanics; Education; Biotransport Phenomena; Bone, Joint and Spine Mechanics; Brain Injury; Cardiac Mechanics; Cardiovascular Devices, Fluids and Imaging; Cartilage and Disc Mechanics; Cell and Tissue Engineering; Cerebral Aneurysms; Computational Biofluid Dynamics; Device Design, Human Dynamics, and Rehabilitation; Drug Delivery and Disease Treatment; Engineered Cellular Environments.* (Sunriver, Oregon, USA, 2013).

[35] Blaber EA (2013). Microgravity induces pelvic bone loss through osteoclastic activity, osteocytic osteolysis, and osteoblastic cell cycle inhibition by CDKN1a/p21. PLoS ONE.

[36] Zhang B, Cory E, Bhattacharya R, Sah R, Hargens AR (2013). Fifteen days of microgravity causes growth in calvaria of mice. Bone.

[37] Blaber EA (2014). Mechanical unloading of bone in microgravity reduces mesenchymal and hematopoietic stem cell-mediated tissue regeneration. Stem Cell Res..

[38] Berg-Johansen B (2016). Spaceflight-induced bone loss alters failure mode and reduces bending strength in murine spinal segments. J. Orthop. Res..

[39] Macaulay TR, Siamwala JH, Hargens AR, Macias BR (2017). Thirty days of spaceflight does not alter murine calvariae structure despite increased Sost expression. Bone Rep..

[40] Gerbaix M (2017). One-month spaceflight compromises the bone microstructure, tissue-level mechanical properties, osteocyte survival and lacunae volume in mature mice skeletons. Sci. Rep..

[41] Gerbaix M (2018). Eight days of earth reambulation worsen bone loss induced by 1-month spaceflight in the major weight-bearing ankle bones of mature mice. Front. Physiol..

[42] Shiba D (2017). Development of new experimental platform ‘MARS’-Multiple Artificial-gravity Research System-to elucidate the impacts of micro/partial gravity on mice. Sci. Rep..

[43] Maupin KA (2019). Skeletal adaptations in young male mice after 4 weeks aboard the International Space Station. NPJ Microgr..

[44] Tominari T (2019). Hypergravity and microgravity exhibited reversal effects on the bone and muscle mass in mice. Sci. Rep..

[45] Cann, C. E., Patterson-Buckendahl, P. & Adachi, R. R. Calcium metabolism and correlated endocrine measurements in primates during Cosmos ‘83. Report No. TM-88223, 129–144 (NASA, 1986).

[46] Cann, C., Rakhmanov, A. & Karolkov, V. *Analysis of Radiographs and Biosamples From Primate Studies*. Report No. TM-102254, 513–519 (NASA, 1990).

[47] Zerath E (1996). Effects of spaceflight on bone mineralization in the rhesus monkey. J. Appl. Physiol..

[48] Zérath E (2002). Spaceflight affects bone formation in rhesus monkeys: a histological and cell culture study. J. Appl. Physiol..

[49] Zérath E, Holy X, Malouvier A, Caissard JC, Noguès C (1991). Rat and monkey bone study in the Biocosmos 2044 space experiment. Physiologist.

[50] Turner RT, Morey ER, Liu C, Baylink DJ (1979). Altered bone turnover during spaceflight. Physiologist.

[51] Wronski TJ, Morey-Holton E, Jee WS (1980). Cosmos 1129: spaceflight and bone changes. Physiologist.

[52] Jee, W. S. S., Kimmel, D. B., Smith, C. & Dell, R. B. *Quantitative Analysis of Selected Bone Parameters: Supplement 2: Bone Elongation Rate and Bone Mass in Metaphysis of Long Bones*. Report No. TM-81289, 149–175 (NASA, 1981).

[53] Spengler DM, Morey ER, Carter DR, Turner RT, Baylink DJ (1983). Effects of spaceflight on structural and material strength of growing bone. Exp. Biol. Med..

[54] Wronski TJ, Morey ER (1983). Effect of spaceflight on periosteal bone formation in rats. Am. J. Physiol..

[55] Wronski TJ, Morey ER (1983). Alterations in calcium homeostasis and bone during actual and simulated space flight. Med. Sci. Sports Exerc..

[56] Doty SB (1985). Morphologic and histochemical studies of bone cells from SL-3 rats. Physiologist.

[57] Vico L (1987). Effects of weightlessness on bone mass and osteoclast number in pregnant rats after a five-day spaceflight (COSMOS 1514). Bone.

[58] Holton, E., Berretta, D., Doty, S., Roberts, W. & Garetto, L. Gravity and Skeletal Growth: I. Gravity and Skeletal Growth. Report No. TM-102254, 113–122 (NASA, 1990).

[59] Morey-Holton ER, Arnaud SB (1991). Skeletal responses to spaceflight. Adv. Space Biol. Med..

[60] Rakhmanov AS (1991). The state of bone tissue in monkeys in experiments in the Cosmos-1887 biosatellite]. Kosm. Biol. Aviakosm Med..

[61] Vico L, Novikov VE, Very JM, Alexandre C (1991). Bone histomorphometric comparison of rat tibial metaphysis after 7-day tail suspension vs. 7-day spaceflight. Aviat. Space Environ. Med..

[62] Kaplansky AS, Durnova GN, Burkovskaya TE, Vorotnikova EV (1991). The effect of microgravity on bone fracture healing in rats flown on Cosmos-2044. Physiologist.

[63] Doty SB, Morey-Holton ER, Durnova GN, Kaplansky AS (1992). Morphological studies of bone and tendon. J. Appl. Physiol..

[64] Kirchen ME (1995). Effects of microgravity on bone healing in a rat fibular osteotomy model. Clin. Orthop. Relat. Res.

[65] Durnova G, Kaplanskii A, Morey-Holton E, Vorobéva V (1996). [Investigation of tibial bones of the rats exposed on board ‘Spacelab-2’: Histomorphometric analysis]. Kosm. Biol. Aviakosm Med..

[66] Cavolina J (1997). The effects of orbital spaceflight on bone histomorphometry and messenger ribonucleic acid levels for bone matrix proteins and skeletal signaling peptides in ovariectomized growing rats 1. Endocrinology.

[67] Zerath E (2000). Effects of Bion 11 14-day space flight on monkey iliac bone. J. Gravit. Physiol..

[68] Doty SB (2004). Space flight and bone formation. Materwiss Werksttech.

[69] Johnson RB (2005). Effects of spaceflight on the attachment of tendons to bone in the hindlimb of the pregnant rat. Anat. Rec..

[70] Tavella S (2012). Bone turnover in wild type and pleiotrophin-transgenic mice housed for three months in the International Space Station (ISS). PLoS ONE.

[71] Keune JA, Philbrick KA, Branscum AJ, Iwaniec UT, Turner RT (2016). Spaceflight-induced vertebral bone loss in ovariectomized rats is associated with increased bone marrow adiposity and no change in bone formation. NPJ Microgr..

[72] Dadwal UC (2019). The effects of spaceflight and fracture healing on distant skeletal sites. Sci. Rep..

[73] Mikolajewicz, N. & Komarova, S. V. Meta-analytic methodology for basic research: a practical guide. *Front. Physiol*. **10**, 203 (2019). 10.3389/fphys.2019.00203 PMC6445886 30971933

[74] Oganov VS (2005). [Reactions of the human bone system in space flight: phenomenology]. Kosm. Biol. Aviakosm Med..

[75] Vico L (2017). Cortical and trabecular bone microstructure did not recover at weight-bearing skeletal sites and progressively deteriorated at non-weight-bearing sites during the year following international space station missions. J. Bone Miner. Res..

[76] Lang T (2004). Cortical and trabecular bone mineral loss from the spine and hip in long-duration spaceflight. J. Bone Miner. Res..

[77] Cunningham HC (2018). Age-dependent bone loss and recovery during hindlimb unloading and subsequent reloading in rats. BMC Musculoskelet. Disord..

[78] Ishijima M (2001). Enhancement of osteoclastic bone resorption and suppression of osteoblastic bone formation in response to reduced mechanical stress do not occur in the absence of osteopontin. J. Exp. Med..

[79] Morse A (2014). Mechanical load increases in bone formation via a sclerostin-independent pathway. J. Bone Miner. Res..

[80] Basso N, Bellows CG, Heersche JNM (2005). Effect of simulated weightlessness on osteoprogenitor cell number and proliferation in young and adult rats. Bone.

[81] David V (2006). Two-week longitudinal survey of bone architecture alteration in the hindlimb-unloaded rat model of bone loss: sex differences. Am. J. Physiol. Endocrinol. Metab..

[82] Bloomfield SA, Allen MR, Hogan HA, Delp MD (2002). Site- and compartment-specific changes in bone with hindlimb unloading in mature adult rats. Bone.

[83] Chen MM (1992). Adaptation of cancellous bone to aging and immobilization in growing rats. Anat. Rec..

[84] Barou O, Palle S, Vico L, Alexandre C, Lafage-Proust MH (1998). Hindlimb unloading in rat decreases preosteoblast proliferation assessed in vivo with BrdU incorporation. Am. J. Physiol. Endocrinol. Metab..

[85] Shen V (1997). Short-term immobilization-induced cancellous bone loss is limited to regions undergoing high turnover and/or modeling in mature rats. Bone.

[86] Marenzana M, Arnett TR (2013). The key role of the blood supply to bone. Bone Res..

[87] Leblanc AD, Schneider VS, Evans HJ, Engelbretson DA, Krebs JM (1990). Bone mineral loss and recovery after 17 weeks of bed rest. J. Bone Miner. Res..

[88] Ronca AE (2019). Behavior of mice aboard the International Space Station. Sci. Rep..

[89] Corlett T, Stavnichuk M, Komarova SV (2020). Population analysis of space travelers. Life Sci. Space Res..

[90] Stanley TD, Doucouliagos H (2015). Neither fixed nor random: weighted least squares meta-analysis. Stat. Med..

[91] Hedges LV (1983). A random effects model for effect sizes. Psychol. Bull..

