# Supplementary material for: Bone health in spacefaring rodents and primates: systematic review and meta-analysis
Source: NPJ Microgravity. 2021 Jun 1;7:19. doi: 10.1038/s41526-021-00147-7 (PMC8169759; doi:10.1038/s41526-021-00147-7)

<u>Supplementary Information</u> for the manuscript "Bone health in spacefaring rodents and primates: systematic review and meta-analysis" by Jingyan Fu, Matthew Goldsmith, Sequoia D. Crooks, Sean F. Condon, Martin Morris, Svetlana V. Komarova

<u>Supplementary notes</u>

Supplementary note 1. Search Strategy

1. Animal*.mp or exp Mammals/ or mammal.mp.
2. exp Hominidae/ or Hominidae.mp. or exp Pan troglodytes/ or exp Pongidae/ or chimpanzee.mp. or exp Hylobates/ or gibbon.mp. or ape.mp or great ape.mp
3. exp Cercopithecidae/ or Monkey.mp. or Macaca.mp. or Macaque.mp. or exp Macaca mulatta/ or rhesus monkey.mp. or exp Macaca fascicularis/ or cynomolgus monkey.mp. or crab eating macaque.mp. or exp Macaca nemestrina/ or pig-tailed macaque.mp. or pigtail macaque.mp
4. exp Saimiri/ or squirrel monkey.mp.
5. exp Cebus/ or Cebus paella.mp or tufted capuchin.mp.
6. exp Rodentia/ or rodent.mp. or exp Muridae/or exp Cricetidae/ or exp Rats/ or rat*.mp. or rattus.mp. or exp Mice/ or mouse.mp. or mice.mp or mus.mp or exp Gerbillinae/ or Meriones.mp. or Gerbil*.mp. or exp Caviidae/ or exp Guinea Pigs/ or Guinea pig.mp.
7. exp Lagomorph*/ or exp Leporidae/ or exp Rabbits/ or rabbit*.mp.
8. exp Carnivor*/ or exp Canidae/ or exp Dogs/ or dog*.mp. or Canis.mp. or Canidae.mp. or exp Felidae/ or Cat.mp. or felis.mp.
9. exp Reptiles/ or exp Chelon*/ or exp Turtles/ or Tortoise.mp. or Turtle.mp. or Reptilia.mp. or exp Lizards/ or Gecko.mp. or Sauria.mp.
10. exp Birds/ or exp Chickens/ or exp Quail/ or exp Galliformes/ or exp Coturnix/ or Bird.mp. or chick*.mp. or quail.mp. or galliform*.mp. or Coturnix.mp. or Gallus.mp.
11. exp Amphibians/ or Amphibi*.mp. or exp Anura/ or Salientia.mp. or anura.mp or exp Ranidae/ or frog.mp. or rana.mp. or exp Bufonidae/ or toad.mp. or Bufo.mp or exp Xenopus/ or Xenopus.mp. or exp Urodela/ or Urodela.mp. or Newt.mp. or Salamander.mp. or Urodel*.mp.
12. exp Pisces/ or exp Osteichthyes/ or Fish.mp. or exp Fish/ or Oryzias.mp. or Medaka.mp. or Zebrafish.mp. or Danio.mp. or Xiphophorus.mp. or Mummichog.mp. or Cyprinus.mp.
13. or/1-12
14. exp Space Flight/
15. exp Weightlessness/
16. exp Extraterrestrial Environment/
17. ((soyuz* or apollo* or gemini or "international space station" or saluyt or skylab or shenzhou or voskhod or euromir or NASA or voskhod or tiangong or mir or mercury or shuttle or ISS or ESA or CNSA or NASDA or Sputnik* or Atlas or Biosatellite or Zond or Bion or Spacelab or Foton or Genesis or SpaceX) and (space* or orbit* or station* or mission*)).ti,ab,kf.
18. (space adj5 (flight* or travel* or explor* or outer)).ti,ab,kw.
19. or/14-18
20. exp "Bone and Bones"/ or exp Bone Diseases/ or exp Osteogenesis/ or exp Bone Density/ or exp Bone Remodeling/
21. (bone* or osseo* or osteo* or skelet* or musculoskelet*).ti,ab,kw.
22. (skeletal or musculoskeletal or tarsal* or metatarsal* or calcaneus or talus or femur or fibula or patella or fibia or humerus or radius or ulna or clavicle or acromion or glenoid or diaphyses or epiphyses or hyoid or sesamoid or cranium or cranial or occipital or basilar or foramen or basicranium or sphenoid or mastoid or petrous or odontoid or parietal or fossa or skull or spenoid

or mandible or maxilla or vomer or zygoma or vertebra* or sacrum or rib or ribs or sternum or manubrium or coccyx).ti,ab,kw.
23. or/20-22
24. 13 and 19 and 23

Supplementary note 2. Quality score checklist for full text appraisal

(Total of 25)

1. Mission title & flight duration are clearly stated (1)
2. Clear indication of: (maximum of 4)
   sex (1), age (1), weight preflight (0.5), weight postflight (0.5), and sample size: n_{SF} (1) of spaceflight animals
3. Study contains the following control groups: (maximum 3 points)
   preflight or baseline control group (1), ground control group (1), vivarium control group (1)
4. Specify housing conditions of: (maximum 2 points)
   spaceflight group: group vs single housing (0.5) and specific habitat (0.5);
   ground control group: specific conditions in reference to spaceflight group (1)
5. Time of sacrifice/measurements for: (maximum 2 points)
   spaceflight group (1) and control group(s) (1)
6. All data was presented in a table (1) or in a graph form (0);
7. When averaged data are presented clearly show sample size (n) for each measurement (2)
8. Clearly indicate all measurement units (including type of data spread) (2)
9. Specific bone region from which measurements are taken is defined (1) and measurement techniques used are indicated (1)
10. Data regarding the following bone parameters shown: (maximum 3 points)
    trabecular bone parameters (1), cortical bone parameters (1), bone turnover parameters (1)
11. Accurately report appropriate data/units (3), or contains evidence of misreporting (0)

Supplementary Tables

Supplementary Table 1. PRISMA Checklist

Section/topic	#	Checklist item	Reported on page #
**TITLE**			
Title	1	Identify the report as a systematic review, meta-analysis, or both.	1
**ABSTRACT**			
Structured summary	2	Provide a structured summary including, as applicable: background; objectives; data sources; study eligibility criteria, participants, and interventions; study appraisal and synthesis methods; results; limitations; conclusions and implications of key findings; systematic review registration number.	2
**INTRODUCTION**			
Rationale	3	Describe the rationale for the review in the context of what is already known.	3
Objectives	4	Provide an explicit statement of questions being addressed with reference to participants, interventions, comparisons, outcomes, and study design (PICOS).	3
**METHODS**			
Protocol and registration	5	Indicate if a review protocol exists, if and where it can be accessed (e.g., Web address), and, if available, provide registration information including registration number.	The study was not registered
Eligibility criteria	6	Specify study characteristics (e.g., PICOS, length of follow-up) and report characteristics (e.g., years considered, language, publication status) used as criteria for eligibility, giving rationale.	17-18
Information sources	7	Describe all information sources (e.g., databases with dates of coverage, contact with study authors to identify additional studies) in the search and date last searched.	16-17
Search	8	Present full electronic search strategy for at least one database, including any limits used, such that it could be repeated.	Supplemental information S1
Study selection	9	State the process for selecting studies (i.e., screening, eligibility, included in systematic review, and, if applicable, included in the meta-analysis).	17,Supplemental information S2
Data collection process	10	Describe method of data extraction from reports (e.g., piloted forms, independently, in duplicate) and any processes for obtaining and confirming data from investigators.	17
Data items	11	List and define all variables for which data were sought (e.g., PICOS, funding sources) and any assumptions and simplifications made.	Table 1, S2
Risk of bias in individual studies	12	Describe methods used for assessing risk of bias of individual studies (including specification of whether this was done at the study or outcome level), and how this information is to be used in any data synthesis.	21
Summary measures	13	State the principal summary measures (e.g., risk ratio, difference in means).	18-20
Synthesis of results	14	Describe the methods of handling data and combining results of studies, if done, including measures of consistency (e.g., I^2) for each meta-analysis.	18-20

Risk of bias across studies	15	Specify any assessment of risk of bias that may affect the cumulative evidence (e.g., publication bias, selective reporting within studies).	21
Additional analyses	16	Describe methods of additional analyses (e.g., sensitivity or subgroup analyses, meta-regression), if done, indicating which were pre-specified.	21-22
**RESULTS**			
Study selection	17	Give numbers of studies screened, assessed for eligibility, and included in the review, with reasons for exclusions at each stage, ideally with a flow diagram.	4-5, Fig. 1A, Table S3
Study characteristics	18	For each study, present characteristics for which data were extracted (e.g., study size, PICOS, follow-up period) and provide the citations.	Table 2, S4, S5
Risk of bias within studies	19	Present data on risk of bias of each study and, if available, any outcome level assessment (see item 12).	Fig. 2, S1, S2
Results of individual studies	20	For all outcomes considered (benefits or harms), present, for each study: (a) simple summary data for each intervention group (b) effect estimates and confidence intervals, ideally with a forest plot.	Fig. 3-7, Tables S6-S18
Synthesis of results	21	Present results of each meta-analysis done, including confidence intervals and measures of consistency.	6-9, Fig. 3-7, Table 3, S6-S18
Risk of bias across studies	22	Present results of any assessment of risk of bias across studies (see Item 15).	Fig. 2, S1
Additional analysis	23	Give results of additional analyses, if done (e.g., sensitivity or subgroup analyses, meta-regression [see Item 16]).	9-10, Fig. 2, 8, S2-S7
**DISCUSSION**			
Summary of evidence	24	Summarize the main findings including the strength of evidence for each main outcome; consider their relevance to key groups (e.g., healthcare providers, users, and policy makers).	10-14
Limitations	25	Discuss limitations at study and outcome level (e.g., risk of bias), and at review-level (e.g., incomplete retrieval of identified research, reporting bias).	14-15
Conclusions	26	Provide a general interpretation of the results in the context of other evidence, and implications for future research.	15-16
**FUNDING**			
Funding	27	Describe sources of funding for the systematic review and other support (e.g., supply of data); role of funders for the systematic review.	22

Supplementary Table 2. Alternative Terms used for included parameters

Parameters (Abbreviation)	Alternate Term
**Trabecular Bone Measures**	
1. Trabecular bone volume fraction (Tb.BV/TV)	- BV/TV [27,28,31]<br>- Trabecular Bone Volume [15,18,19,27,28,31]<br>- Cancellous Bone Volume [24,28,30]<br>- Fractional Area of Mineralized Tissue [15]
2. Trabecular Number (Tb.N)	- Trabecular density [18]
3. Trabecular Thickness (Tb.Th)	N/A
4. Trabecular Separation (Tb.Sp)	N/A
5. Connectivity Density	N/A
6. Total BV/TV	- Total Bone Volume [27]
**Cortical Bone Measures**	
1. Marrow Area (Ma.Ar)	- Medullary Area [11,12,17,23,32]
2. Marrow Diameter (Ma.Dm)	- Medullary Diameter [21]
3. Cortical Bone Area (Ct.Ar)	- Cortical Plate Area [17]<br>- Cortical Cross-sectional area [23]
4. Cortical Thickness (Ct.Th)	- Cortical Width [28]
**Bone Turnover Measures**	
Osteoblast Surface (Ob.S)	-Forming surface [12]
Osteoblast Number (N.Ob)	- Osteoblast number per bone perimeter (Ob.N/B.Pm) [19]<br>- Number of osteoblasts per length of bone [15,19]
Osteoid Surface (OS/BS)	- Length of osteoid seam covering bone forming Surfaces [24,28]<br>- percentage of bone area covered in osteoid [27]
Osteoid Thickness (O.Th)	- Thickness of osteoid seam [18]<br>- width of osteoid surface [27]
Osteoclast Surface (Oc.S)	- Active Resorption Surface [18,24]<br>- Fraction of bone surface length covered with Osteoclasts [18,24,27,28]
Osteoclast Number (N.Oc.)	- Osteoclast number per bone perimeter (N.Oc./B.Pm) [28]<br>- Number of osteoclasts per length of bone [18,19,24,28]
Bone Formation Rate (BFR)	N/A
Mineral Apposition Rate (MAR)	- Calcification rate [49]

BV/TV = Bone volume/tissue volume

Supplementary Table 3. Removed Articles with Quantitative measures of bone health

Article Reference	Mission(s)	Species	Exclusion Reason
**Turner 1979 et al. [50]**	Cosmos 782 & 936	Rats	Only show relative changes, raw data present in Morey 1978 (Cosmos 782) and Morey-Holton 1978 (Cosmos 936)
**Wronski 1980 et al. [51]**	Cosmos 1129	Rats	Data present Wronski 1981 and Jee 1983
**Jee 1981 et al. [52]**	Cosmos 1129	Rats	Data present in Jee 1983
**Spengler 1983 et al. [53]**	Cosmos 936	Rats	Data present in Morey-Holton 1978
**Wronski 1983a et al. [55]**	Cosmos 782, 936, & 1129	Rats	Data present in Morey 1978 (Cosmos 782), Morey-Holton 1978 (Cosmos 936), and Wronski 1981 (Cosmos 1129)
**Wronski 1983b et al. [54]**	Cosmos 1129	Rats	Data present in Wronski 1981
**Doty 1985 [56]**	SpaceLab 3	Rats	Measure of osteoblast number did not have a defined location
**Vico 1987 et al. [57]**	Cosmos 1514	Rats	All spaceflight rodents were pregnant
**Holton 1990 et al. [58]**	Cosmos 1887	Rats	Data present in Doty 1990
**Morey-Holton 1991 et al. [59]**	Cosmos 936	Rats	Data present in Morey-Holton 1978
**Rakhmanov 1991 et al. [60]**	Cosmos 1887	Primates	Data present in Cann 1990
**Vico 1991 et al. [61]**	Cosmos 1667	Rats	Data present in Vico 1988
**Kaplansky 1991 et al. [62]**	Cosmos 2044	Rats	All spaceflight animals received bone fracture
**Doty 1992 et al. [63]**	Cosmos 2044	Rats	Measured number of "active" osteoblasts, not included due to vague definition of active
**Kirchen 1995 et al. [64]**	STS-29	Rats	Measure osteoclast number per arbitrary bone region
**Durnova 1996 et al. [65]**	STS-58	Rats	All data presented as averages with no measure of variation for any recorded parameter
**Cavolina 1997 et al. [66]**	STS-62	Rats	All spaceflight animals were ovariectomized
**Zerath 2000b et al. [67]**	Bion 11	Primates	Data presented better in Zerath 2002
**Doty 2004 [68]**	Cosmos 1129	Rats	Data presented in Wronski 1981
**Johnson 2005 et al. [69]**	STS-66	Rats	All spaceflight rodents were pregnant
**Tavella 2012 et al. [70]**	MDS	Mice	Only contain 3 spaceflight mice, all but 1 died before returning to Earth
**Keune 2016 et al.[71]**	STS-62	Rats	All spaceflight animals were ovariectomized
**Dadwal 2019 et al. [72]**	SpaceX CRS-10	Mice	Data present in Maupin 2019

Supplementary Table 4. Parameters including in meta-analysis

Year	Mission	Article References	Species	Tb.BV/TV	Tb.Th	Tb.N	Tb.Sp	Con.D	T.BV/TV	Ob.S	N.Ob	OS/BS	O.Th	Oc.S	N.Oc	Ma.A/D	Ct.Ar	Ct.Th	BFR	MAR
1975	Cosmos 782	**Asling 1978** [10]	**Rats**	✓	✓	✓														
		**Morey 1978 et al.** [11]														✓			✓	
1977	Cosmos 936	**Morey-Holton 1978 et al.** [12]	**Rats**							✓					✓	✓			✓	
1979	Cosmos 1129	**Judy 1981** [13]	**Rats**				✓													
		**Wronski 1981 et al.** [14]														✓				
		**Jee 1983 et al.** [15]		✓							✓									
		**Rogacheva 1984 et al.** [16]														✓		✓		
1983	Cosmos 1514	**Cann 1986 et al.** [45]	**Primates**															✓		
1985	Cosmos 1667	**Kaplanskii 1987 et al.** [17]	**Rats**	✓							✓				✓	✓	✓			
		**Vico 1988 et al.** [18]		✓	✓	✓	✓					✓	✓	✓	✓					
1985	SpaceLab 3	**Wronski 1987 et al.** [19]	**Rats**	✓						✓	✓			✓	✓				✓	
1987	Cosmos 1887	**Doty 1990 et al.** [20]	**Rats**													✓	✓			
		**Vailas 1990 et al.** [21]														✓	✓			
		**Zerath 1990 et al.** [22]		✓																
		**Cann 1990 et al.** [46]	**Primates**															✓		
1989	Cosmos 2044	**Zérath 1991 et al.** [49]	**Primates**	✓						✓										
			**Rats**																	✓
		**Vailas 1992 et al.** [23]														✓	✓			
		**Vico 1993 et al.** [24]		✓	✓	✓	✓			✓		✓		✓	✓					
1992	Bion 10	**Zerath 1996b et al.** [47]	**Primates**	✓	✓	✓	✓													✓
1992	STS-52	**Turner 1995 et al.** [25]	**Rats**	✓		✓	✓			✓		✓		✓	✓					
1992	STS-57	**Westerlind 1995 et al.** [26]	**Rats**	✓												✓	✓		✓	✓
1993	STS-58 (SLS-2)	**Zerath 1996a et al.** [27]	**Rats**	✓	✓	✓	✓		✓	✓		✓	✓	✓						
		**Lafage-Proust 1998 et al.** [28]		✓	✓	✓				✓		✓		✓	✓					
1996	Bion 11	**Zerath 2002 et al.** [48]	**Primates**	✓	✓	✓	✓					✓	✓	✓				✓	✓	✓
1996	STS-77	**Bateman 1998 et al.** [29]	**Rats**																	✓
1996	STS-78	**Wronski 1998 et al.** [30]	**Rats**	✓						✓				✓			✓	✓	✓	✓
		**Zerath 2000a et al.** [31]		✓	✓	✓	✓			✓			✓						✓	
		**Vajda 2001 et al.** [32]														✓	✓		✓	✓

Year	Mission	Article Reference	Species	Tb.BV/TV	Tb.Th	Tb.N	Tb.Sp	Con.D	T.BV/TV	Ob.S	N.Ob	OS/BS	O.Th	Oc.S	N.Oc	Ma.A/D	Ct.Ar	Ct.Th	BFR	MAR
2001	STS-108	**Lloyd 2015 et al.** [33]	**Mice**	✓	✓	✓	✓	✓		✓				✓				✓	✓	✓
2007	STS-118	**Ortega 2013 et al.** [34]	**Mice**	✓	✓	✓	✓	✓									✓			✓
2010	STS-131	**Blaber 2013 et al.** [37]	**Mice**						✓					✓	✓					
		**Zhang 2013 et al.** [36]							✓											
		**Blaber 2014 et al.** [37]		✓	✓	✓	✓	✓								✓	✓	✓		
2013	Bion M1	**Berg-Johansen 2016 et al.** [38]	**Mice**	✓	✓															
		**Macaulay 2017 et al.** [39]							✓									✓		
		**Gerbaix 2017 et al.** [40]		✓	✓	✓	✓	✓		✓		✓		✓		✓	✓	✓		
		**Gerbaix 2018 et al.** [41]							✓									✓		
2016	SpaceX CRS-9	**Shiba 2017 et al.** [42]	**Mice**	✓																
2017	SpaceX CRS-10	**Maupin 2019 et al.** [43]	**Mice**	✓	✓	✓	✓		✓							✓	✓	✓		
2017	SpaceX CRS-12	**Tominari 2019 et al.** [44]	**Mice**	✓	✓	✓	✓													

Parameter abbreviations: Tb.BV/TV = trabecular BV/TV; Tb.Th = trabecular thickness; Tb.N = trabecular number; Tb.Sp = trabecular separation; Con.D = connective density; T.BV/TV = total BV/TV; Ob.S = osteoblast surface area; N.Ob = osteoblast number; OS/BS = osteoid surface/bone surface; O.Th = osteoid thickness; Oc.S = osteoclast surface area; N.Oc = osteoclast number; Ma.A/D = bone marrow area/diameter; Ct.A = cortical bone area; Ct.Th = cortical bone thickness; BFR = bone formation rate; MAR = mineral apposition rate.

Supplementary Table 5. Rodent study characteristics used for covariate analysis

Mission	Articles	Bones (Sub-sections)			Species	Strain	Sex	Age	SF Sacrifice Delay	Group House	Sham Op.	GC Cond. (scale 1-3)
		Region 1	Region 2	Region 3								
Cosmos 782	**Asling 1978**			Tibia (M)	Rats	Wistar	Male	9w	NR			3
	**Morey 1978**			Tibia (D)								
Cosmos 936	**Morey-Holton 1978**			Tibia (D)	Rats	Wistar	Male	9w	NR			3
Cosmos 1129	**Judy 1981**			Tibia (M)	Rats	Wistar	Male	11w 6d	7-11h			3
	**Wronski 1981**	Rib (NS)	Humerus (D)	Tibia (D)								
	**Jee 1983**		Humerus (M)	Tibia (M)								
	**Rogacheva 1984**		Femur (D)									
Cosmos 1667	**Kaplanskii 1987**	Vertebrae (L)	Pelvis (Ilium)	Tibia (D/M)	Rats	Wistar	Male	15w	4-8h<br>6h			2
	**Vico 1988**	Vertebrae (T8/L1)	Femur (M)	Tibia (M)								
SpaceLab3	**Wronski 1987**	Vertebra (L4)	Humerus (M)	Tibia (D)	Rats	Sprague-Dawley	Male	(S): 8w<br>(L): 12w	11-17h			NR
Cosmos 1887	**Vailas 1990**		Humerus (D)		Rats	Wistar	Male	12w 6d	1d 18h	✓		3
	**Doty 1990**			Tibia (D)								
	**Zerath 1990**	Vertebrae (NS)	Humerus (M)									
Cosmos 2044	**Zerath 1991**	Vertebra (T9)	Humerus (M)		Rats	Wistar	Male	12w 5d	3-11h	✓	✓	3
	**Vailas 1992**		Humerus (D)									
	**Vico 1993**	Vertebrae (L2/T5)	Femur (M)	Tibia (E/M)								
STS-52	**Turner 1995**		Humerus (M)		Rats	Sprague-Dawley	Male	6w	2d 5h	✓		NR
STS-57	**Westerlind 1995**		Femur (D)	Tibia (M)	Rats	Fischer 344	Male	7-8w	5-8h	✓		2
STS-58<br>(SLS-2)	**Zerath 1996a**	Vertebrae (T9/C7)	Humerus (M)		Rats	Sprague-Dawley	Male	8w	4-6h			2
	**Lafage-Proust 1998**		Femur (M),<br>Humerus (M)									

STS-77	**Bateman 1998**		Humerus (D)	Tibia (D)	Rats	Sprague-Dawley	Male	5w 5d	3-6h	✓		N/A
STS-78	**Wronski 1998**	Vertebra (L1)		Tibia (M/D)	Rats	Sprague-Dawley	Male	6w 3d	4-7h	✓	✓	1
	**Zerath 2000a**	Vertebra (T8)	Pelvis (Cotyloid)									
	**Vajda 2001**		Femur (D)									
STS-108	**Lloyd 2015**	Vertebra (L5)	Humerus (M)<br>Femur (D)	Tibia (M)	Mice	C57BL/6	Female	9w 1d	3h 30m	✓		2
STS-118	**Ortega 2013**		Femur (M)	Tibia (M/D)	Mice	C57BL/6	Female	9w	3-6h	✓		NR
STS-131	**Blaber 2013**		Pelvis (Ischium),<br>Femur (M)		Mice	C57BL/6J	Female	16w	2h	✓		1
	**Zhang 2013**	Calvaria										
	**Blaber 2014**		Femur (E/M)									
Bion M1	**Berg-Johansen 2016**	Vertebrae (C)			Mice	C57BL/6N	Male	19-20w	13-15h<br>13-24h	✓		2
	**Macaulay 2017**	Calvaria										
	**Gerbaix 2017**	Vertebrae (L1/L3/T12)	Femur (M/D)									
	**Gerbaix 2018**			Calcaneus,<br>Navicular,<br>Talus								
SpaceX CRS-9	**Shiba 2017**		Femur (prox)		Mice	C57BL/6J	Male	8w	NR			3
SpaceX CRS-10	**Maupin 2019**	Calvaria, Rib (10),<br>Sternum, Vertebra (L4)	Humerus(M/D),<br>Femur (M/D)	Tibia (M/D)	Mice	C57BL/6J	Male	9w	NR	✓	✓	2
SpaceX CRS-12	**Tominari 2019**		Humerus (prox)	Tibia (prox)	Mice	C57BL/6J	Male	9w	NR			3

Bones are organized by skeletal region (Region 1: bones of the head, vertebrae and thorax, Region 2: pelvis, humerus and femur, Region 3: tibia and ankle). Longbone sub-sections (epiphysis (E), metaphysis (M), or diaphysis (D)) are indicated. For vertebrae type (lumber (L), thoracic (T), or caudal (C)) and number are indicated. w = weeks, h = hours. m = minutes. NS = not specified. NR = not recorded. GC Cond. = Ground control conditions rated from 1 (poorest) to 3 (best) consideration to spaceflight associated conditions other than microgravity.

Supplementary Table 6. Trabecular Number

				SF vs GC		GC vs VC	
**Species**	**Flight**	**Days**	**n_{SF}/n_{GC}**	**ES (%)**	**95% CI**	**ES (%)**	**95% CI**
Mice	STS-108	12	12	3.8	[-1.3,9.0]	NA	NA
	STS-118	13	12	-8.5	[-14.0,-3.1]	NA	NA
	STS-131	15	8	-6.0	[-10.8,-1.2]	NA	NA
	Bion M1 (2)	30	5/6	-10.2	[-22.6,2.3]	-9.2	[-18.7,0.2]
	SpaceX CRS-10	28	10	-0.5	[-17.9,16.9]	NA	NA
	SpaceX CRS-12	34	3	-43.7	[-71.7,-15.8]	NA	NA
**Mice Overall**				**-6.2**	**[-15.9,3.6]**	NA	NA
Rats	Cosmos 782	19.5	6	-24.7	[-42.7,-6.7]	8.7	[-8.5,26.0]
	Cosmos 1667	7	7	-22.0	[-43.8,-0.2]	NA	NA
	Cosmos 2044	14	5	-1.8	[-16.4,12.9]	-1.2	[-34.4,32.1]
	STS-52	10	6	8.4	[-14.8,31.6]	NA	NA
	STS-58	14	5	-11.1	[-29.7,7.5]	11.9	[-8.0,31.8]
	STS-78	17	6	-4.9	[-15.1,5.2]	6.8	[-4.2,17.9]
**Rats Overall**				**-9.9**	**[-19.2,-0.5]**	NA	NA
**Rodents Overall**				**-7.8**	**[-15.4,-0.1]**	**3.3**	**[-9.2,15.7]**
				**I^2 = 68.8**		**I^2 = 52.1**	

Supplementary Table 7. Trabecular Separation

				SF vs GC		GC vs VC	
**Species**	**Flight**	**Days**	**n_{SF}/n_{GC}**	**ES (%)**	**95% CI**	**ES (%)**	**95% CI**
Mice	STS-108	12	12	-4.0	[-9.8,1.8]	NA	NA
	STS-118	13	12	10.4	[3.3,17.5]	NA	NA
	STS-131	15	8	2.6	[-3.4,8.6]	NA	NA
	Bion M1 (2)	30	5/6	11.4	[-3.7,26.5]	14.6	[1.7,27.5]
	SpaceX CRS-10	28	10	0.5	[-11.5,12.4]	NA	NA
	SpaceX CRS-12	34	3	110.9	[19.7,202.0]	NA	NA
**Mice Overall**				**10.4**	**[-13.8,34.7]**	NA	NA
Rats	Cosmos 1129*	18.5	7	3.4	[-17.9,24.7]	NA	NA
	Cosmos 1667	7	7	56.4	[10.6,102.1]	NA	NA
	Cosmos 2044	14	5	10.4	[-14.7,35.6]	7.2	[-42.5,56.9]
	STS-52	10	6	-16.8	[-52.9,19.4]	NA	NA
	STS-58	14	5	25.6	[-6.2,57.5]	-7.8	[-39.1,23.5]
	STS-78	17	6	7.0	[-6.8,20.7]	-5.7	[-19.2,7.9]
**Rats Overall**				**15.0**	**[-2.2,32.2]**	NA	NA
**Rodents Overall**				**12.4**	**[-4.8,29.6]**	**2.3**	**[-17.1,21.7]**
				**I^2 = 80.8**		**I^2 = 42.8**	

Supplementary Table 8. Connective Density

Species	Flight	Days	n_{SF}	SF vs GC ES (%)	SF vs GC 95% CI
Mice	STS-108	15	12	-10.2	[-41.8,21.4]
	STS-118	12	12	-45.0	[-67.3,-22.6]
	STS-131	13	8	13.5	[-6.4,33.3]
	Bion M1 (2)	30	5	-58.0	[-126.8,10.7]
**Mice Overall**				**-22.8**	**[-64.7,19.0]**
				I^2 **= 83.5**	

Supplementary Table 9. Total BV/TV

Species	Flight	Days	n_{SF}	SF vs GC ES (%)	SF vs GC 95% CI
Mice	STS-131	15	8	1.2	[-4.4,6.8]
	Bion M1 (1)	30	6	-5.0	[-27.5,17.5]
	Bion M1 (2)	30	5	-2.2	[-7.3,2.9]
	SpaceX CRS-10	30	10	1.9	[1.2,2.6]
**Mice Overall**				**-0.5**	**[-7.9,6.9]**
Rats	STS-58	14	5	-4.6	[-14.3,5.2]
**Rodents Overall**				**-1.1**	**[-9.1,6.9]**
				I^2 **= 94.5**	

Supplementary Table 10. Osteoblast Surface

Species	Flight	Days	n_{SF}/n_{GC}	SF vs GC ES (%)	SF vs GC 95% CI	GC vs VC ES (%)	GC vs VC 95% CI
Mice	STS-108	12	12	-15.4	[-99.5,68.5]	*NA*	*NA*
	Bion M1 (2)	30	5/6	-35.2	[-82.1,11.7]	-11.5	[-63.1,40.1]
Rats	Cosmos 936	18.5	5	12.1	[-4.3,28.5]	*NA*	*NA*
	SpaceLab 3 (L)	7	4/5	-33.3	[-79.9,13.3]	*NA*	*NA*
	SpaceLab 3 (S)	7	6	-4.2	[-35.0,26.6]	*NA*	*NA*
	Cosmos 2044	14	5	-25.7	[-173.1,121.8]	-9.2	[-164.7,146.4]
	STS-58	14	5	-19.4	[-45.0,6.3]	-10.0	[-43.4,23.4]
	STS-78	17	6	-20.3	[-48.5,7.9]	1.5	[-31.1,34.1]
**Rats Overall**				**-15.2**	**[-56.4,25.9]**	*NA*	*NA*
**Rodents Overall**				**-17.4**	**[-54.3,19.5]**	**-6.9**	**[-20.2,6.4]**
				I^2 **= 50.8**		I^2 **= 0**	

Supplementary Table 11. Osteoblast Number

				SF vs GC		GC vs VC	
**Species**	**Flight**	**Days**	**n_{SF}/n_{GC}**	**ES (%)**	**95% CI**	**ES (%)**	**95% CI**
Rats	Cosmos 1129	18.5	7	-36.0	[-66.5,-5.5]	0	[-21.0,21.0]
	Cosmos 1667	7	7	-16.4	[-23.7,-9.2]	-8.1	[-15.1,-1.2]
	SpaceLab 3 (L)	7	4/5	-33.9	[-78.6,10.8]	*NA*	*NA*
	SpaceLab 3 (S)	7	6	-8.9	[-42.2,24.4]	*NA*	*NA*
**Rats Overall**				**-23.2**	**[-51.0,4.7]**	**-4.1**	**[-9.0,0.8]**
				**I^2 = 71.9**		**I^2 = 31.7**	

Supplementary Table 12. Osteoid Surface

				SF vs GC		GC vs VC	
**Species**	**Flight**	**Days**	**n_{SF}/n_{GC}**	**ES (%)**	**95% CI**	**ES (%)**	**95% CI**
Mice	Bion M1 (2)	30	5/6	4.7	[-28.3,37.6]	-25.4	[-47.6,-3.1]
Rats	Cosmos 1667	7	7	-33.7	[-51.2,-16.2]	*NA*	*NA*
	Cosmos 2044	14	5	-18.2	[-37.6,1.2]	-29.3	[-44.9,-13.6]
	STS-52	10	6	-79.7	[-113.6,-45.9]	*NA*	*NA*
	STS-58	14	5	-11.0	[-40.8,18.8]	*NA*	*NA*
**Rodents Overall**				**-29.9**	**[-53.9,-5.8]**	**-27.1**	**[-29.8,-24.5]**
				**I^2 = 74.5**		**I^2 = 0**	

Supplementary Table 13. Osteoid Thickness

				SF vs GC		GC vs VC	
**Species**	**Flight**	**Days**	**n_{SF}/n_{GC}**	**ES (%)**	**95% CI**	**ES (%)**	**95% CI**
Rats	Cosmos 1667	7	7	-16.7	[-27.0,-6.4]	*NA*	*NA*
	STS-58	14	5	-24.6	[-54.0,4.8]	-24.1	[-54.8,6.6]
	STS-78	17	6	-45.9	[-89.9,-2.0]	6.16	[-35.7,48.1]
**Rats Overall**				**-28.6**	**[-54.5,-2.7]**	**-7.6**	**[-40.2,25.0]**
				**I^2 = 65.8**		**I^2 = 34.3**	

Supplementary Table 14. Bone Marrow Area

				SF vs GC		GC vs VC	
**Species**	**Flight**	**Days**	**n_{SF}/n_{GC}**	**ES (%)**	**95% CI**	**ES (%)**	**95% CI**
Mice	STS-131	15	8	35.9	[-4.5,76.2]	*NA*	*NA*
	Bion M1 (2)	30	5/6	-1.1	[-5.6,3.5]	5.8	[0.1,11.4]
	SpaceX CRS-10	28	10	-3.3	[-18.1,11.4]	*NA*	*NA*
**Mice Overall**				**10.8**	**[-0.2,21.8]**	*NA*	*NA*
Rats	Cosmos 782	19.5	6	-10.4	[-20.5,-0.4]	0.0	[-6.9,6.9]
	Cosmos 936	18.5	4	-8.5	[-17.4,0.0]	10.4	[-2.8,23.7]

	Cosmos 1129#	18.5	6	-3.3	[-30.1,23.4]	-24.5	[-36.8,-12.1]
	Cosmos 1667	7	7	0.0	[-6.6,6.6]	-11.1	[-15.5,-6.7]
	Cosmos 1887#	12.5	5	-7.9	[-31.8,15.9]	1.3	[-8.0,10.7]
	Cosmos 2044	14	5	-5.3	[-15.2,4.6]	2.7	[-2.7,8.1]
	STS-57	11	12	2.6	[-3.3,8.6]	1.0	[-1.5,3.5]
	STS-78	17	6	15.2	[-1.6,32.0]	15.2	[6.8,23.6]
**Rats Overall**				**-1.3**	**[-7.7,5.1]**	**-0.9**	**[-8.9,7.1]**
**Rodents Overall**				**2.4**	**[-4.3,9.1]**	**-0.2**	**[-8.9,8.6]**
				***I^2* = 21.1**		***I^2* =87.3**	

Supplementary Table 15. Cortical Bone Area

				**SF vs GC**		**GC vs VC**	
**Species**	**Flight**	**Days**	**n_{SF}/n_{GC}**	**ES (%)**	**95% CI**	**ES (%)**	**95% CI**
Mice	STS-118	13	9/11	-3.8	[-7.1,-0.4]	*NA*	*NA*
	STS-131	15	8	-7.7	[-13.3,-2.1]	*NA*	*NA*
	Bion M1(2)	30	5/6	-5.7	[-13.7,2.3]	-4.3	[-10.4,1.7]
	SpaceX CRS-10	28	10	-6.4	[-16.9,4.1]	*NA*	*NA*
**Mice Overall**				**-5.9**	**[-8.8,-3.0]**	*NA*	*NA*
Rats	Cosmos 1887	12.5	5	-10.0	[-26.4,6.4]	5.8	[-12.6,24.3]
	Cosmos 2044	14	5	-7.9	[-12.8,-3.0]	-3.1	[-7.9,1.7]
	STS-57	11	12	-6.1	[-9.4,-2.9]	-3.9	[-6.7,-1.0]
	STS-78	17	6	-0.8	[-4.5,2.9]	3.6	[-3.8,11.0]
**Rats Overall**				**-6.0**	**[-9.4,-2.6]**	*NA*	*NA*
**Rodents Overall**				**-5.9**	**[-8.0,-3.8]**	**-1.1**	**[-6.2,4.0]**
				***I^2* =0**		***I^2* = 46.7**	

Supplementary Table 16. Cortical Thickness

				**SF vs GC**		**GC vs VC**	
**Species**	**Flight**	**Days**	**n_{SF}/n_{GC}**	**ES (%)**	**95% CI**	**ES (%)**	**95% CI**
Mice	STS-108	12	12	-10.8	[-19.5,-2.1]	*NA*	*NA*
	STS-131	15	8	-1.4	[-3.6,0.8]	*NA*	*NA*
	Bion M1 (1)	30	6/7	3.0	[-6.5,12.5]	*NA*	*NA*
	Bion M1 (2)	30	5/6	-5.5	[-17.7,6.7]	-3.6	[-12.3,5.0]
	SpaceX CRS-10	28	10	-3.6	[-10.5,3.3]	*NA*	*NA*
**Mice Overall**				**-4.6**	**[-10.8,1.7]**	*NA*	*NA*
Rats	Cosmos 1129	18.5	6	-8.3	[-9.3,-7.3]	-7.2	[-19.9,5.5]
	STS-78	17	6	-1.7	[-6.4,2.9]	4.8	[-0.8,10.4]
**Rodents Overall**				**-4.7**	**[-13.7,4.4]**	**-2.0**	**[-11.8,7.7]**
				***I^2* = 90.7**		***I^2* = 68.6**	

Supplementary Table 17. Bone Formation Rate

				SF vs GC		GC vs VC	
**Species**	**Flight**	**Days**	**n_{SF}/n_{GC}**	**ES (%)**	**95% CI**	**ES (%)**	**95% CI**
Mice	STS-108	12	12	-71.2	[-86,9,-55.5]	*NA*	*NA*
Rats	Cosmos 782	19.5	11/7	-40.5	[-53.1,-27.9]	1.3	[-9.9,12.4]
	Cosmos 936	18.5	10/8	-17.3	[-40.4,5.8]	9.6	[-16.3,35.4]
	Cosmos 1129	18.5	11	-33.7	[-46.9,-20.5]	-10.5	[-36.3,15.4]
	SpaceLab 3 (L)	7	4	-33.7	[-65.1,-2.3]	*NA*	*NA*
	STS-57	11	12	-10.6	[-33.6,12.4]	-12.8	[-30.2,4.6]
	STS-78	17	6	3.2	[-25.0,31.3]	1.7	[-27.0,30.4]
**Rats Overall**				**-22.8**	**[-39.1,-6.6]**	*NA*	*NA*
**Rodents Overall**				**-31.6**	**[-50.4,-12.8]**	**-9.2**	**[-24.3,5.8]**
				**I^2 = 83.8**		**I^2 = 65.4**	

Supplementary Table 18. Mineral Apposition Rate

				SF vs GC		GC vs VC	
**Species**	**Flight**	**Days**	**n_{SF}/n_{GC}**	**ES (%)**	**95% CI**	**ES (%)**	**95% CI**
Mice	STS-108	12	12	-30.4	[-46.0,-14.7]	*NA*	*NA*
	STS-118	13	9/11	-22.2	[-46.7,2.2]	*NA*	*NA*
Rats	STS-57	11	12	-2.5	[-24.0,19.0]	-2.6	[-20.9,25.8]
	STS-77*	10	6/8	-1.9	[-25.9,22.2]	*NA*	*NA*
	STS-78	17	6	-0.1	[-21.7, 21.5]	5.0	[-14.3,24.4]
**Rats Overall**				**-8.9**	**[-28.1,10.3]**	*NA*	*NA*
**Rodents Overall**				**-13.5**	**[-27.1,0.1]**	**-0.1**	**[-38.8,38.7]**
				**I^2 = 51.9**		**I^2 = 0**	

Days = mission duration; n_{SF} = spaceflight animal group sample size; n_{GC} = ground control sample size (only indicated if differ from SF group). SF vs GC indicates outcomes of spaceflight to ground control comparisons. GC vs VC indicates outcomes of ground control to vivarium control comparisons. ES (%) = effect size or percent difference; 95% CI = 95% confidence interval.

* = Mission outcomes where GC not present, and a VC is used as the comparison control

# = contains measures of bone marrow area derived from marrow diameter

Supplementary Figures

Supplementary Figure 1. Heterogeneity and sensitivity analyses for Tb.N. a, b) Heterogeneity was analyzed using single mission exclusion (a) and cumulative mission exclusion (b). Red area: 95% CI for the global effect size (left axis); line: I^2 (right axis). c) Funnel plot; d) article-level standard error $SE\ (\theta_P)$ as a function of quality score. R^2 and p-value is shown.

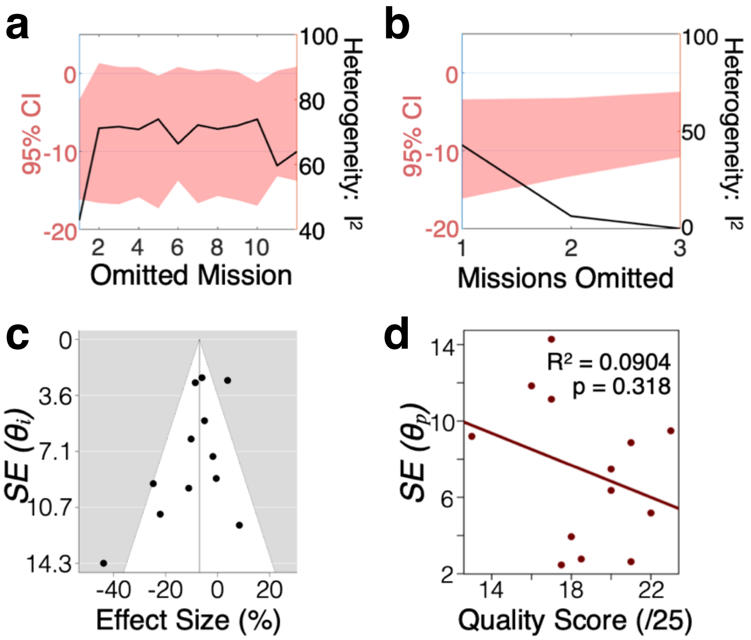

Supplementary Figure 2. Sub-group analysis of reported outcome by paper quality score. Rodent paper-level outcomes were divided into two groups, with quality score ≥ 20 or quality score < 20 on a 25-point scale. N_P is number of papers-level outcomes. Square/line: overall effect size (ES(%)) and 95% CI, numerical values of each are presented on the right. * indicate parameters for which the subgroups differ in their statistical significance from zero.

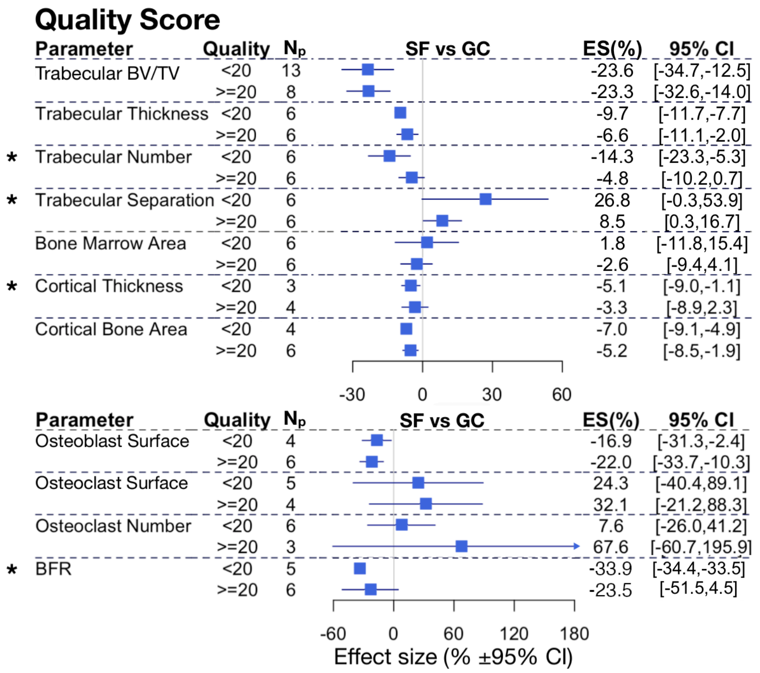

Supplementary Figure 3. Animal related subgroups. Rodent mission-level outcomes divided by animal age (a), sex (b), and strain (c). (a) Age at launch was <10 weeks (young animals) or ≥ 10 weeks (older animals). (b) Only mice missions were included, as all rat studies were performed exclusively with males. (c) Only rat missions were included for Wistar rats (W) and Sprague-Dawley rats (S-D), as all mice studies were performed with variants of the C57BL/6 lineage. N is number of mission-level outcomes in each sub-group. Square/line: overall effect size (ES(%)) and 95% CI, numerical values of each are presented on the right. * indicate parameters for which the subgroups differ in their statistical significance from zero. ** indicate parameters in which subgroups are significantly different from one another.

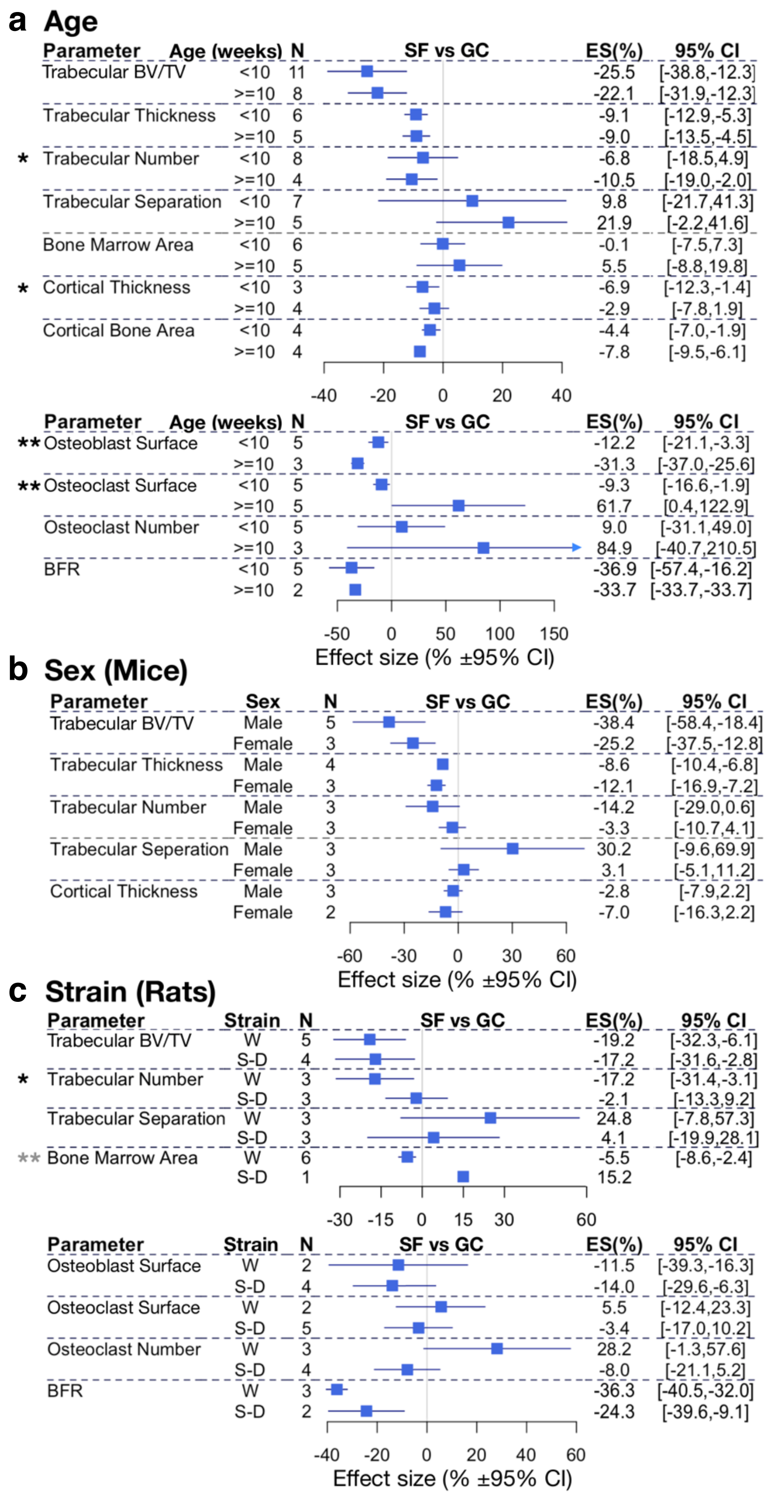

Supplementary Figure 4. Mission related subgroups. Rodent mission-level outcomes divided by mission-duration (a), single vs grouped housing condition (b), and space agency (c). (a) Mission duration subgroups: short duration missions (<14 days); long duration missions (>= 14 days). (b) Housing condition subgroups: animals housed as a group (Yes) and those individually housed (No). (c) Space agency subgroups: NASA, Roscosmos, and JAXA. N is number of mission-level outcomes in each sub-group. Square/line: overall effect size (ES(%)) and 95% CI, numerical values of each are presented on the right. * indicate parameters for which the subgroups differ in their statistical significance from zero. ** indicate parameters in which subgroups are significantly different from one another.

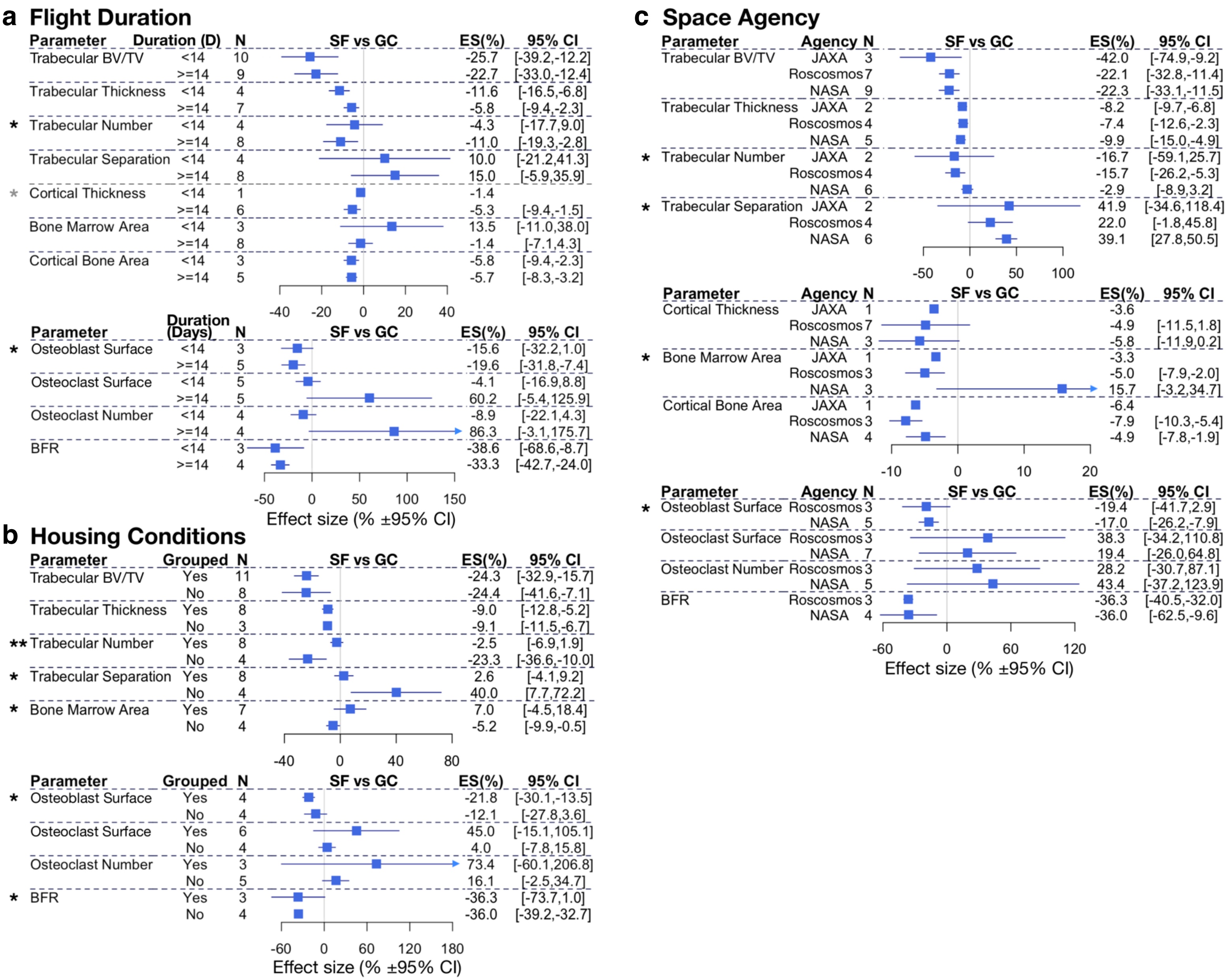

Supplementary Figure 5. Study related subgroups. Rodent mission-level outcomes divided by presence of sham operation (a) sacrifice delay (b), and ground control conditions (c). (a) Sham operation subgroups: missions that report performing sham operations (Yes); mission that report no sham operations (No). (b) Sacrifice subgroups: within 10 h of landing (<10) and longer than 10 h after landing (>=10). (c) specific conditions of ground control animals for each subgroup are indicated. N is number of mission-level outcomes in each sub-group. Square/line: overall effect size (ES(%)) and 95% CI, numerical values of each are presented on the right. * indicate parameters for which the subgroups differ in their statistical significance from zero. ** indicate parameters in which subgroups are significantly different from one another.

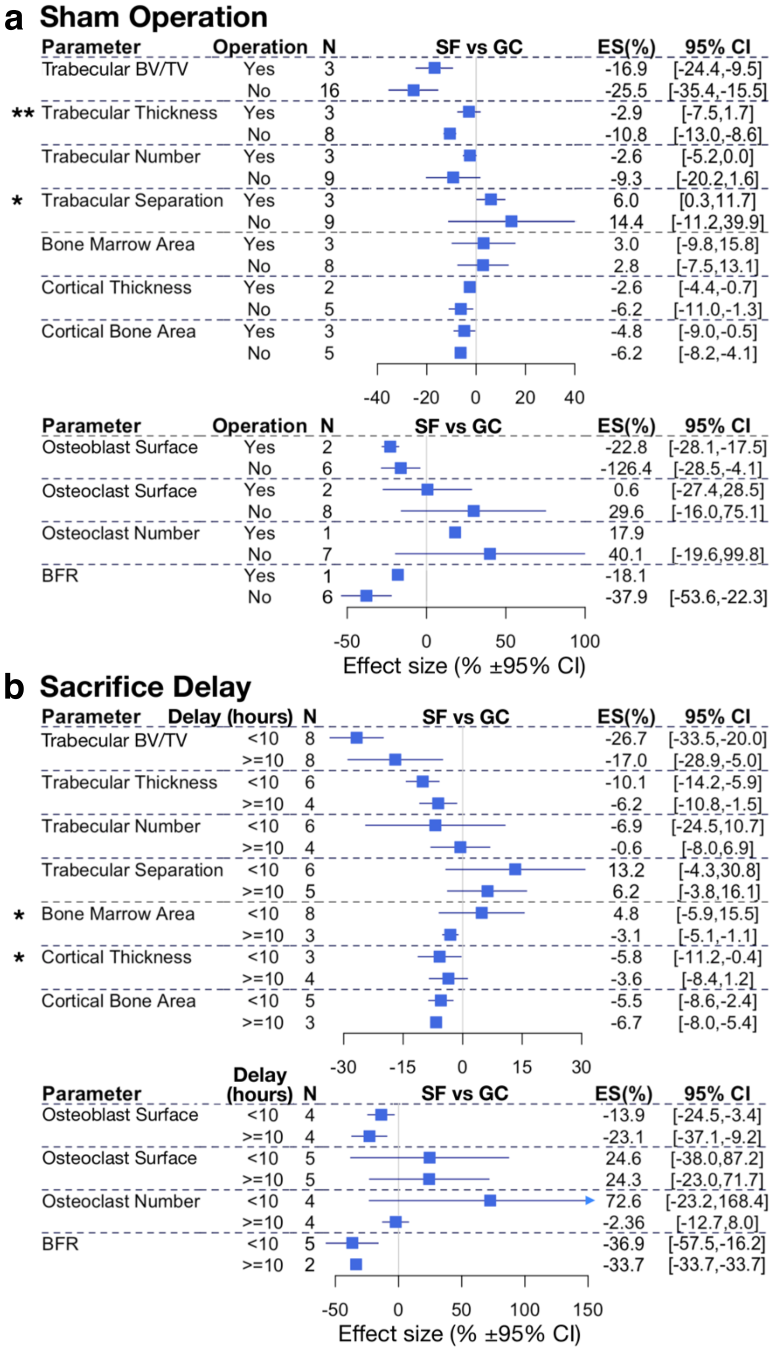

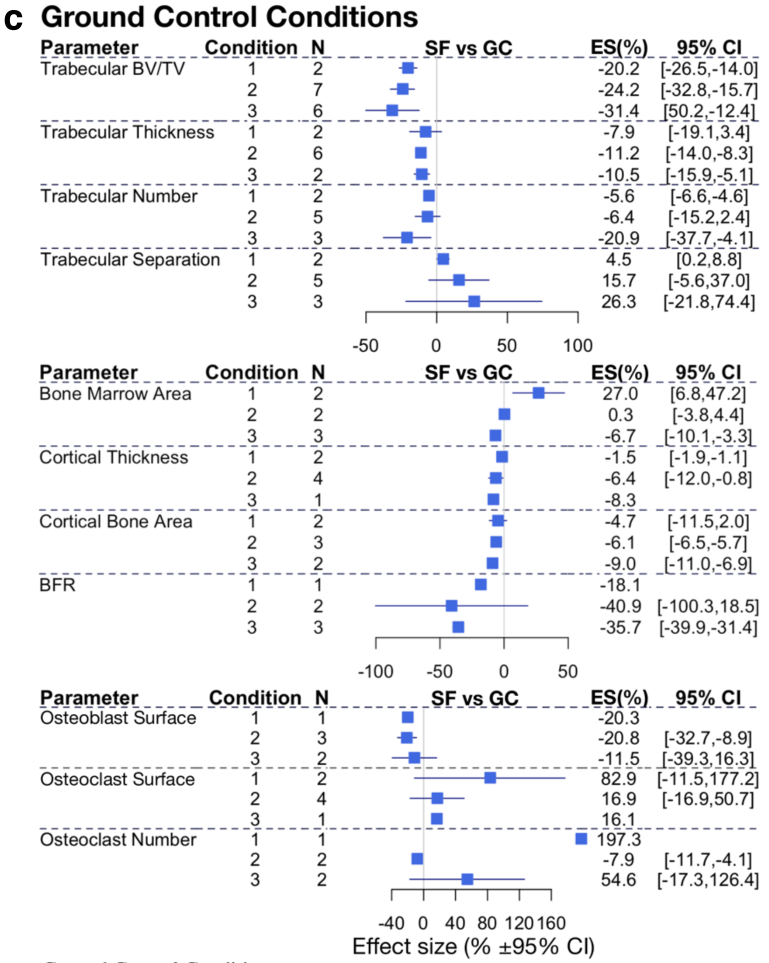

Ground Control Conditions

1. housed in same habitat as the spaceflight group, but not all conditions (e.g. food, and/or temperature, light/dark cycle, etc.) mimicked
2. housed in same habitat as the spaceflight group, and all conditions mimicked, not including force of liftoff or re-entry
3. housed in same habitat as the spaceflight group, and all conditions mimicked, including the force of lift-off and/or re-entry are simulated

Supplementary Figure 6. Meta-regression analysis of sacrifice delay. Mission-level outcomes, measured in effect size (%), of Tb.BV/TV, Ob.S, and Ct.Ar were plotted as a function of sacrifice delay of spaceflight animal group (hours). R^2 is shown. * indicated high R^2.

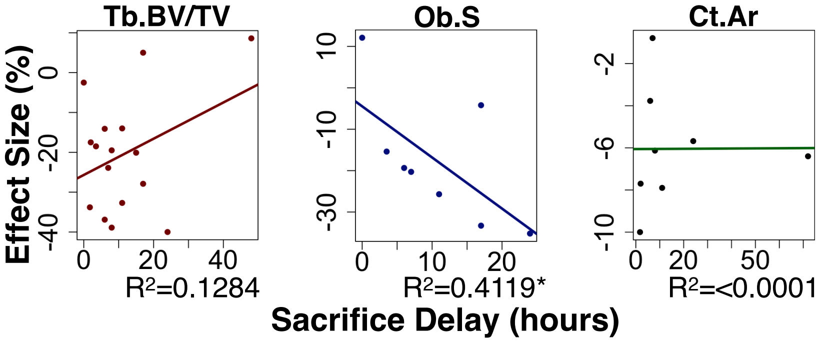

Supplementary Figure 7. Sub-group analysis of bone regions. Rodent bone measurement-level outcomes divided by region: region 1= skull, vertebra, and thorax; region 2 = pelvis, humerus, and femur; region 3 = tibia and ankle bones. N_j is number of bones measured in each sub-group. Square/line: overall effect size (ES(%)) and 95% CI, numerical values of each are presented on the right. * indicate parameters for which the subgroups differ in their statistical significance from zero. ** indicate parameters in which subgroups are significantly different from one another.

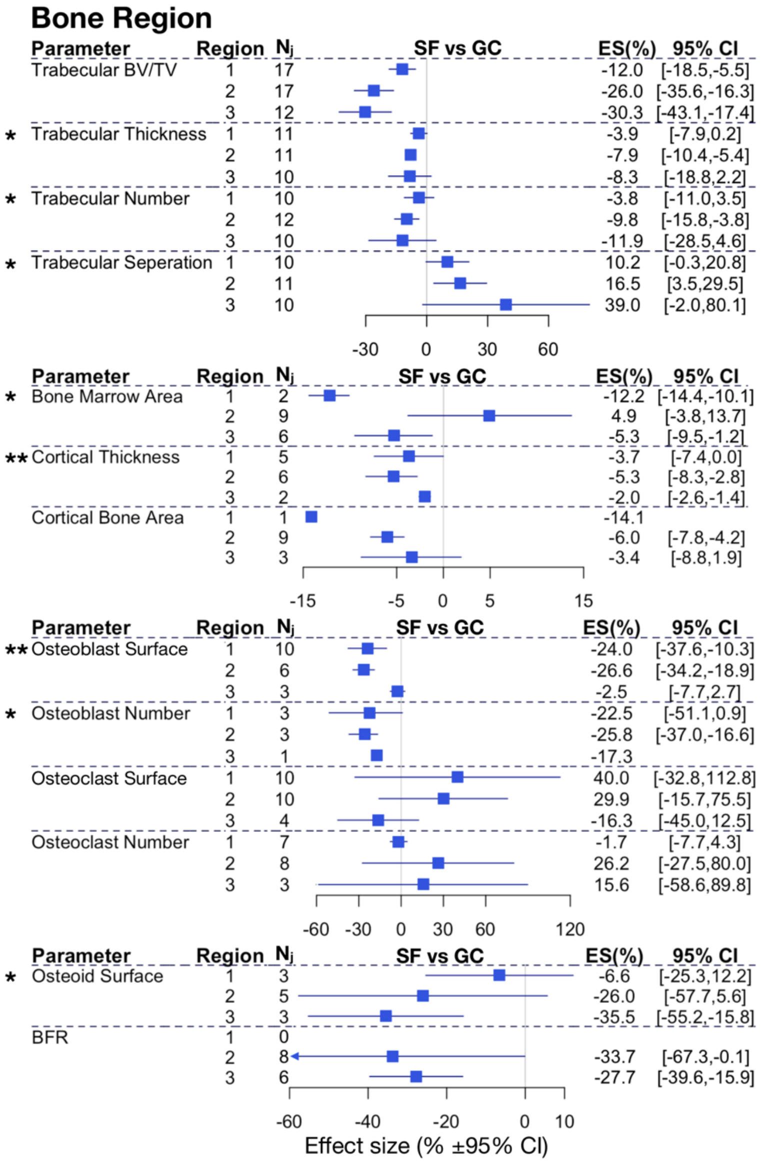

Supplement: Supplementary file 1 — Supplementary Information [file 41526_2021_147_MOESM1_ESM.pdf]
